# Genome-Wide Association Study Reveals Multiple Loci Influencing Normal Human Facial Morphology

**DOI:** 10.1371/journal.pgen.1006149

**Published:** 2016-08-25

**Authors:** John R. Shaffer, Ekaterina Orlova, Myoung Keun Lee, Elizabeth J. Leslie, Zachary D. Raffensperger, Carrie L. Heike, Michael L. Cunningham, Jacqueline T. Hecht, Chung How Kau, Nichole L. Nidey, Lina M. Moreno, George L. Wehby, Jeffrey C. Murray, Cecelia A. Laurie, Cathy C. Laurie, Joanne Cole, Tracey Ferrara, Stephanie Santorico, Ophir Klein, Washington Mio, Eleanor Feingold, Benedikt Hallgrimsson, Richard A. Spritz, Mary L. Marazita, Seth M. Weinberg

**Affiliations:** 1 Department of Human Genetics, Graduate School of Public Health, University of Pittsburgh, Pittsburgh, Pennsylvania, United States of America; 2 Center for Craniofacial and Dental Genetics, Department of Oral Biology, University of Pittsburgh, Pittsburgh, Pennsylvania, United States of America; 3 Department of Pediatrics, Seattle Children’s Craniofacial Center, University of Washington, Seattle, Washington, United States of America; 4 Department of Pediatrics, University of Texas McGovern Medical Center, Houston, Texas, United States of America; 5 Department of Orthodontics, University of Alabama, Birmingham, Alabama, United States of America; 6 Department of Pediatrics, University of Iowa, Iowa City, Iowa, United States of America; 7 Department of Orthodontics, University of Iowa, Iowa City, Iowa, United States of America; 8 Dows Institute, University of Iowa, Iowa City, Iowa, United States of America; 9 Department of Health Management and Policy, University of Iowa, Iowa City, Iowa, United States of America; 10 Department of Biostatistics, University of Washington, Seattle, Washington, United States of America; 11 Human Medical Genetics and Genomics Program, University of Colorado School of Medicine, Aurora, Colorado, United States of America; 12 Department of Mathematical and Statistical Sciences, University of Colorado, Denver, Denver, Colorado, United States of America; 13 Department of Orofacial Sciences, University of California, San Francisco, San Francisco, California, United States of America; 14 Department of Pediatrics, University of California, San Francisco, San Francisco, California, United States of America; 15 Program in Craniofacial Biology, University of California, San Francisco, California, United States of America; 16 Department of Mathematics, Florida State University, Tallahassee, Florida, United States of America; 17 Department of Cell Biology & Anatomy, Cumming School of Medicine, University of Calgary, Calgary, Alberta, Canada; 18 Alberta Children’s Hospital Research Institute, Cumming School of Medicine, University of Calgary, Calgary, Alberta, Canada; 19 McCaig Bone and Joint Institute, Cumming School of Medicine, University of Calgary, Calgary, Alberta, Canada; 20 Department of Pediatrics, University of Colorado School of Medicine, Aurora, Colorado, United States of America; 21 Clinical and Translational Science Institute, School of Medicine, University of Pittsburgh, Pittsburgh, Pennsylvania, United States of America; 22 Department of Psychiatry, School of Medicine, University of Pittsburgh, Pittsburgh, Pennsylvania, United States of America; 23 Department of Anthropology, University of Pittsburgh, Pittsburgh, Pennsylvania, United States of America; Stanford University School of Medicine, UNITED STATES

## Abstract

Numerous lines of evidence point to a genetic basis for facial morphology in humans, yet little is known about how specific genetic variants relate to the phenotypic expression of many common facial features. We conducted genome-wide association meta-analyses of 20 quantitative facial measurements derived from the 3D surface images of 3118 healthy individuals of European ancestry belonging to two US cohorts. Analyses were performed on just under one million genotyped SNPs (Illumina OmniExpress+Exome v1.2 array) imputed to the 1000 Genomes reference panel (Phase 3). We observed genome-wide significant associations (p < 5 x 10^−8^) for cranial base width at 14q21.1 and 20q12, intercanthal width at 1p13.3 and Xq13.2, nasal width at 20p11.22, nasal ala length at 14q11.2, and upper facial depth at 11q22.1. Several genes in the associated regions are known to play roles in craniofacial development or in syndromes affecting the face: *MAFB*, *PAX9*, *MIPOL1*, *ALX3*, *HDAC8*, and *PAX1*. We also tested genotype-phenotype associations reported in two previous genome-wide studies and found evidence of replication for nasal ala length and SNPs in *CACNA2D3* and *PRDM16*. These results provide further evidence that common variants in regions harboring genes of known craniofacial function contribute to normal variation in human facial features. Improved understanding of the genes associated with facial morphology in healthy individuals can provide insights into the pathways and mechanisms controlling normal and abnormal facial morphogenesis.

## Author Summary

There is a great deal of evidence that genes influence facial appearance. This is perhaps most apparent when we look at our own families, since we are more likely to share facial features in common with our close relatives than with unrelated individuals. Nevertheless, little is known about how variation in specific regions of the genome relates to the kinds of distinguishing facial characteristics that give us our unique identities, e.g., the size and shape of our nose or how far apart our eyes are spaced. In this paper, we investigate this question by examining the association between genetic variants across the whole genome and a set of measurements designed to capture key aspects of facial form. We found evidence of genetic associations involving measures of eye, nose, and facial breadth. In several cases, implicated regions contained genes known to play roles in embryonic face formation or in syndromes in which the face is affected. Our ability to connect specific genetic variants to ubiquitous facial traits can inform our understanding of normal and abnormal craniofacial development, provide potential predictive models of evolutionary changes in human facial features, and improve our ability to create forensic facial reconstructions from DNA.

## Introduction

Numerous lines of converging evidence indicate that variation in facial morphology has a strong genetic basis. These include the results of human heritability studies using twin and parent-offspring designs [1–5], Mendelian craniofacial syndromes [6], transgenic animal models with distinctive craniofacial phenotypes [7–9], and studies mapping QTLs for craniofacial shape in several mammalian models [10–14]. However, we still have little understanding of how genetic variation relates to the diversity of normal facial traits commonly observed in humans. Understanding the genetic basis for normal facial variation has important implications for human health. Many genetic syndromes with dysmorphic facies are characterized by relatively subtle morphological changes, often involving quantitative traits with continuous distributions [6]. The range of variation for any given facial trait often displays substantial overlap between affected and healthy individuals. Thus, understanding the genetic factors that contribute to normal facial trait variation may provide valuable insights into the causes of craniofacial dysmorphology, including common craniofacial birth defects such as orofacial clefts [15,16].

To date, only a few studies have explicitly tested for associations between aspects of normal human facial morphology and common genetic variants. Among these, two genome-wide association (GWA) studies have been carried out on healthy individuals of European ancestry using 3D facial imaging and a combination of traditional and more advanced morphometric methods to derive phenotypes [17,18]. Between these two studies, a handful of intriguing genome-wide significant signals were reported, although they were largely non-overlapping. Notably, both studies reported an association between *PAX3* variants and anatomical changes in interorbital region, an intriguing finding given that mutations in *PAX3* cause Waardenburg Syndrome type 1 which is characterized by hypertelorism among other morphological abnormalities. Both studies also reported significant associations with measures of nasal projection in their discovery cohorts, although different genomic regions were implicated. In addition, several more focused candidate gene studies of loci implicated in craniofacial syndromes or in developmental pathways involved in craniofacial development have connected one or more craniofacial dimensions or aspects of shape with a small number of common genetic variants [19–28]. At least three candidate gene studies [20,25,28] have reported modest associations between common variants in *FGFR1* and normal variation in craniofacial morphology, but in each case a different constellation of traits was involved. It is notable that none of the genes from these studies, including *FGFR1*, were identified in the two previous GWA studies of facial morphology.

**Fig 1:**
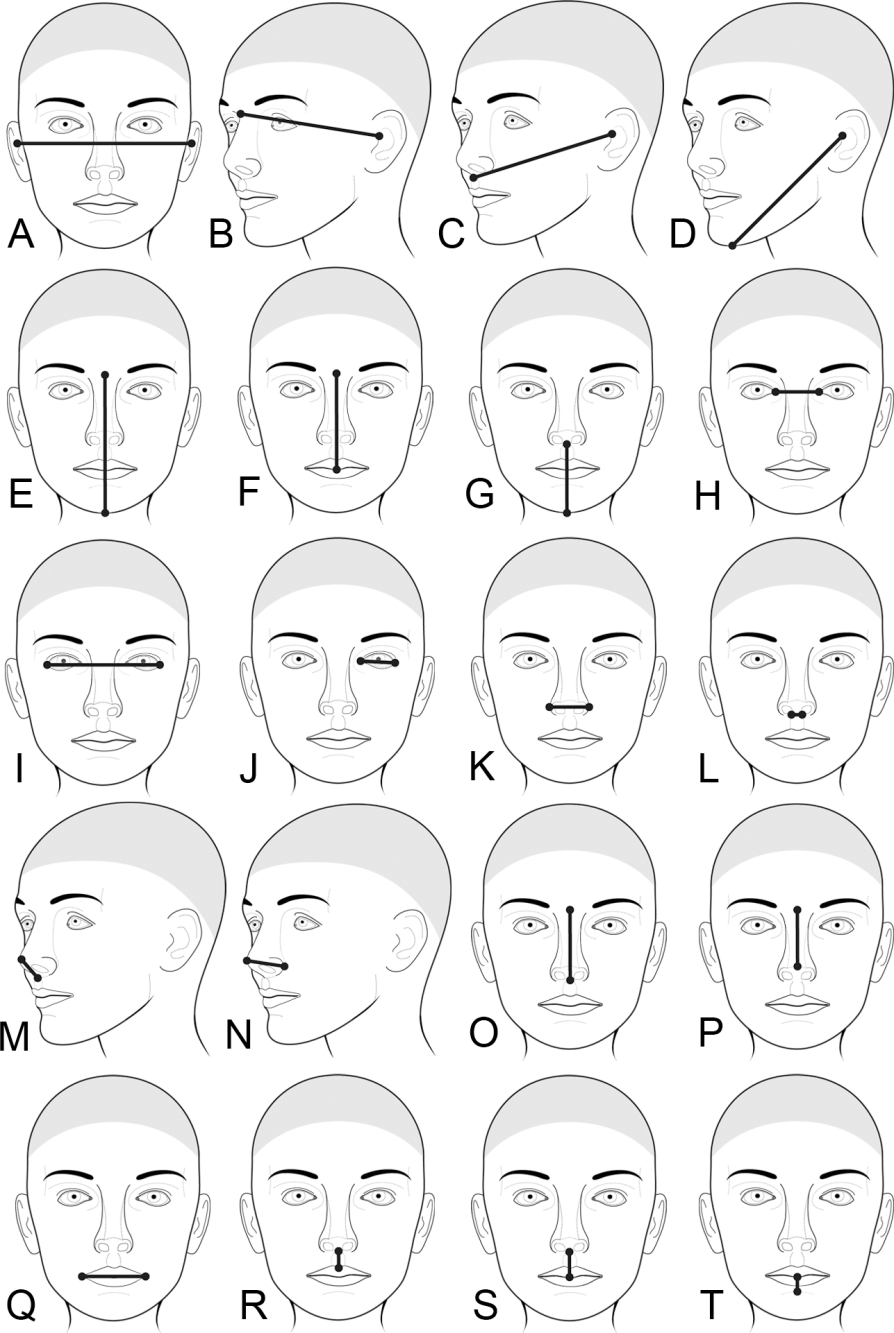
Set of 20 linear distance measurements used in the current study. (A) Cranial base width, (B) Upper facial depth*, (C) Middle facial depth*, (D) Lower facial depth*, (E) Morphological facial height, (F) Upper facial height, (G) Lower facial height, (H) intercanthal width, (I) Outercanthal width, (J) Palpebral fissure length*, (K) Nasal width, (L) Subnasal width, (M) Nasal Protrusion, (N) Nasal ala length*, (O) Nasal height, (P) Nasal Bridge Length, (Q) Labial fissure length, (R) Philtrum length, (S) Upper lip height, and (T) Lower lip height. Measurements with an asterisk (*) are bilateral, but only the left side is shown in the figure.

Thus, while prior studies have detected a handful of biologically plausible genes associated with variation in craniofacial features, it is clear that these efforts are just scratching the surface and the potential for additional discovery is great. In the current study, we performed GWA analyses on a set of 20 craniofacial measurements commonly used in clinical assessment (**Fig 1**) derived from 3D surface images in two well-characterized samples of unrelated White individuals of European ancestry from the USA: a sample comprised of 2447 individuals collected through the University of Pittsburgh (i.e., the Pittsburgh sample) and an independent sample of 671 individuals collected under the direction of the University of Colorado (i.e., the Denver sample). All participants were genotyped using the same SNP array (Illumina OmniExpress+Exome v1.2), which included just under one million SNPs, and were imputed to the 1000 Genomes reference panel (Phase 3). We conducted association tests in each sample separately and combined the results using a meta-analytic approach.

## Results

In total, we observed seven associations in five traits that exceeded the conventional threshold for genome-wide significance (p < 5 x 10^−8^, **Table 1**; **Figs 2–5**). One of the associations also exceeded our study-wide significance threshold of p < 5 x 10^−9^, calculated based on 10 independent traits (see Methods for details). Due to the large number of traits, we will limit our presentation of results to genome-wide significant signals. The entire list of meta-analysis associations with p-values < 5 x 10^−7^ is available in **S1 Table**. Manhattan plots showing the meta-analysis results, as well as the results for each sample, are available in supplemental figures **S1–S20 Figs**.

**Fig 2:**
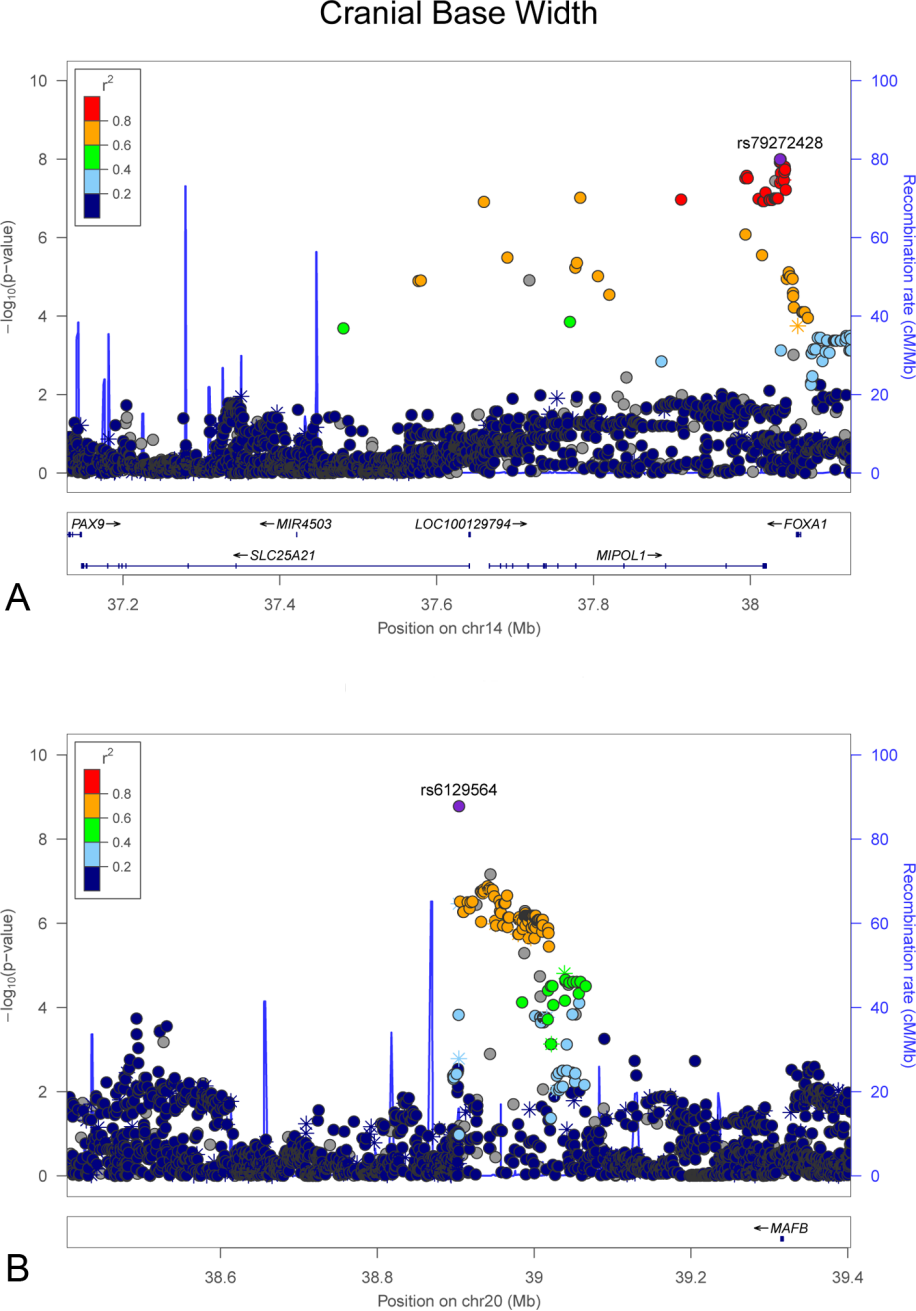
LocusZoom plots showing genome-wide significant associations observed in the meta-analysis for cranial base width (Fig 1A). (A) chromosome 14 and (B) chromosome 20. LocusZoom plots show the association (left y-axis; log10-transformed p-values) with facial traits. Genotyped SNPs are depicted by stars and imputed SNPs are depicted by circles. Shading of the points represent the linkage disequilibrium (r^2^, based on the 1000 Genomes Project Europeans) between each SNP and the top SNP, indicated by purple shading. The blue overlay shows the recombination rate (right y-axis). Positions of genes are shown below the plot.

**Fig 3:**
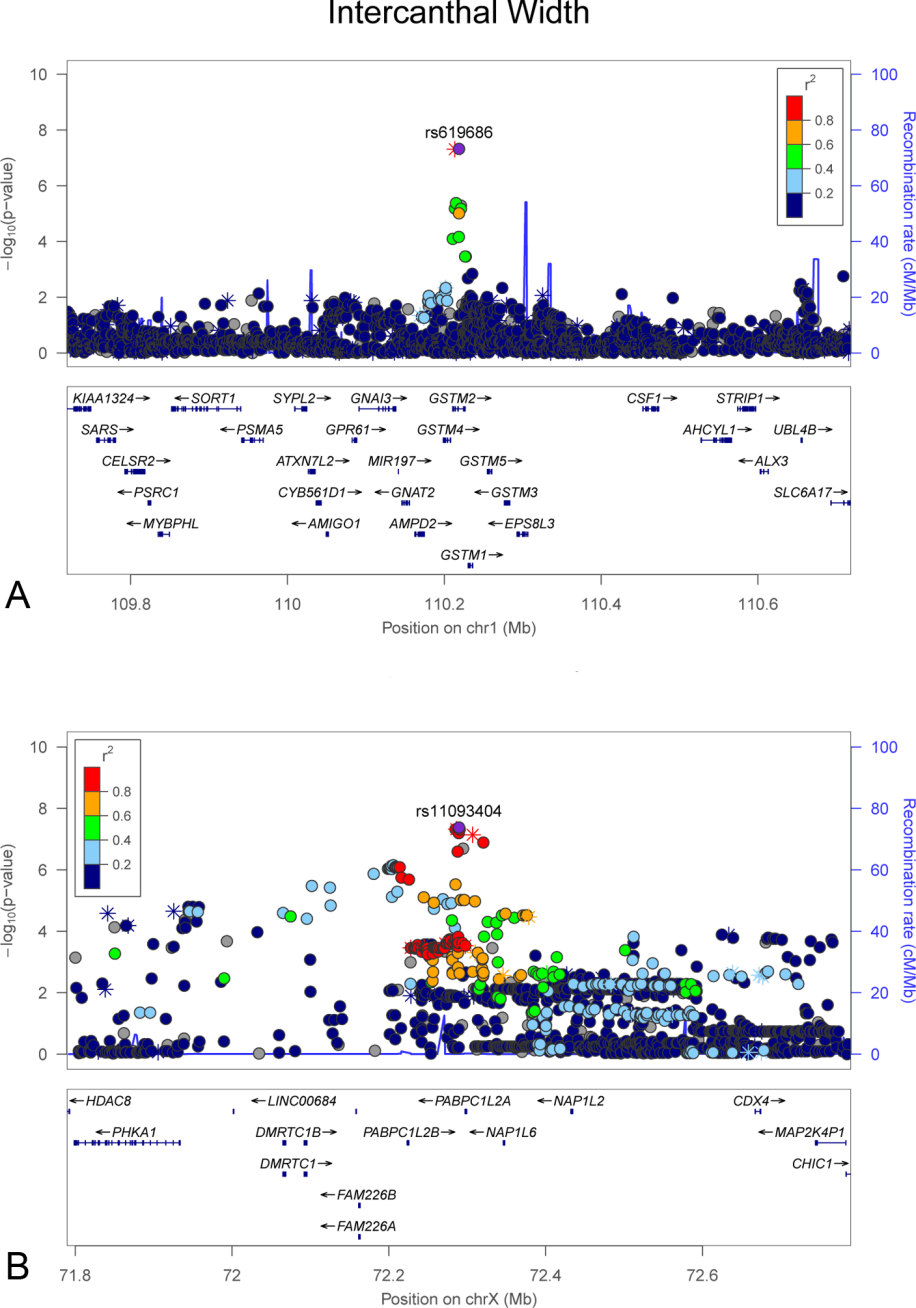
LocusZoom plots showing genome-wide significant associations observed in the meta-analysis for intercanthal width (Fig 1H). (A) chromosome 1 and (B) chromosome X. LocusZoom plots show the association (left y-axis; log10-transformed p-values) with facial traits. Genotyped SNPs are depicted by stars and imputed SNPs are depicted by circles. Shading of the points represent the linkage disequilibrium (r^2^, based on the 1000 Genomes Project Europeans) between each SNP and the top SNP, indicated by purple shading. The blue overlay shows the recombination rate (right y-axis). Positions of genes are shown below the plot.

**Fig 4:**
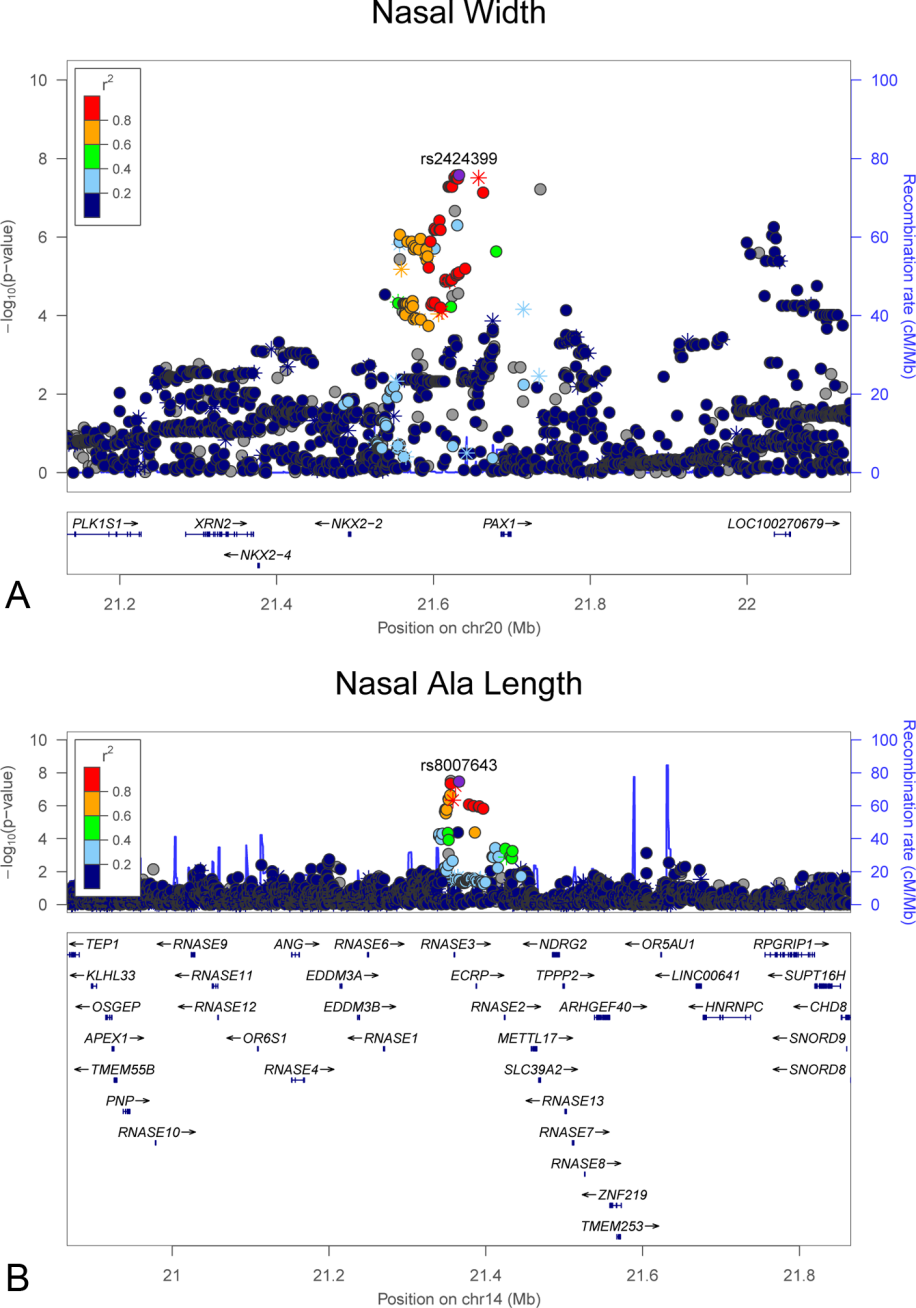
LocusZoom plots showing genome-wide significant associations observed in the meta-analysis for nasal width (Fig 1K) and nasal ala length (Fig 1N). (A) nasal width on chromosome 20 and (B) nasal ala length on chromosome 14. LocusZoom plots show the association (left y-axis; log10-transformed p-values) with facial traits. Genotyped SNPs are depicted by stars and imputed SNPs are depicted by circles. Shading of the points represent the linkage disequilibrium (r^2^, based on the 1000 Genomes Project Europeans) between each SNP and the top SNP, indicated by purple shading. The blue overlay shows the recombination rate (right y-axis). Positions of genes are shown below the plot.

**Fig 5:**
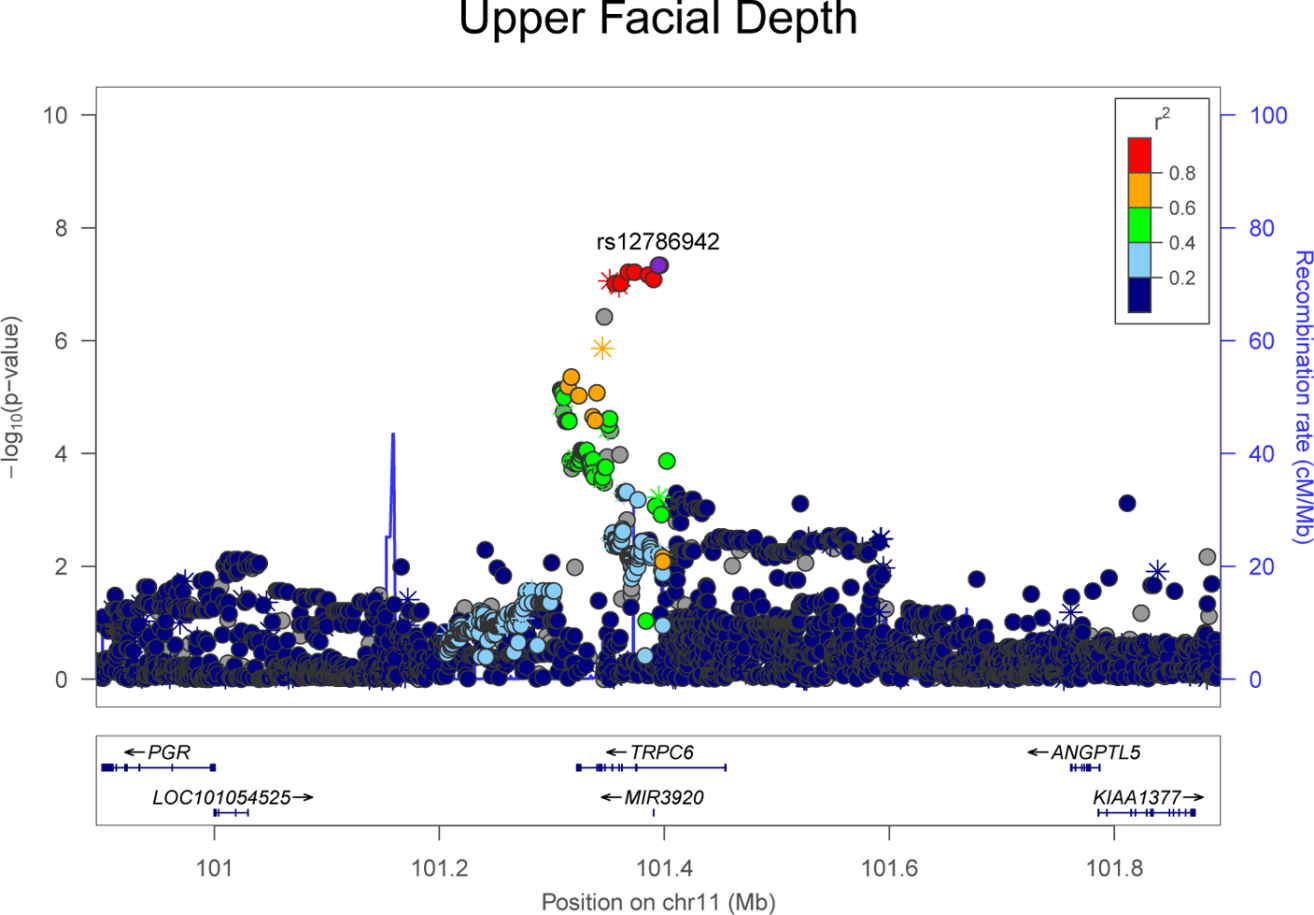
LocusZoom plot showing genome-wide significant association observed in the meta-analysis for upper facial depth (Fig 1B) on chromosome 11. LocusZoom plots show the association (left y-axis; log10-transformed p-values) with facial traits. Genotyped SNPs are depicted by stars and imputed SNPs are depicted by circles. Shading of the points represent the linkage disequilibrium (r^2^, based on the 1000 Genomes Project Europeans) between each SNP and the top SNP, indicated by purple shading. The blue overlay shows the recombination rate (right y-axis). Positions of genes are shown below the plot.

**Table 1:** Genome-wide significant meta-analysis results for five traits.

				Pittsburgh Sample				Denver Sample				Meta-Analysis
Trait	SNP	Locus	Minor allele	MAF	N	Beta (se)^a^	p	MAF	N	Beta (se)^a^	p	p
Cranial base width (Fig 1A)	rs17106852	14q21.1 (38038468)	G	0.106	2368	-1.104 (0.205)	7.91 x 10^−8^	0.097	671	-0.858 (0.406)	3.51 x 10^−2^	**1.01 x 10**^**−8**^
	rs6129564	20q12 (38904203)	A	0.116	2368	-1.210 (0.193)	**4.07 x 10**^**−10**^	0.100	671	-0.449 (0.412)	2.77 x 10^−1^	**1.65 x 10**^**−9**^
Intercanthal width (Fig 1H)	rs619686	1p13.3 (110218761)	G	0.056	2426	-0.763 (0.163)	3.12 x 10^−6^	0.052	671	-0.536 (0.186)	4.12 x 10^−3^	**4.70 x 10**^**−8**^
	rs11093404	Xq13.2 (72289467)	A	0.243	2426	0.427 (0.075)	**1.31 x 10**^**−8**^	0.248	671	0.073 (0.075)	3.32 x 10^−1^	**4.16 x 10**^**−8**^
Nasal width (Fig 1K)	rs2424399	20p11.22 (21632545)	C	0.235	2429	0.377 (0.070)	9.53 x 10^−8^	0.300	671	0.177 (0.098)	7.04 x 10^−2^	**2.62 x 10**^**−8**^
Nasal ala length (Fig 1N)	rs8007643	14q11.2 (21365801)	T	0.067	2426	1.064 (0.186)	**1.19 x 10**^**−8**^	0.069	671	0.221 (0.216)	3.07 x 10^−1^	**3.36 x 10**^**−8**^
Upper facial depth (Fig 1B)	rs12786942	11q22.1 (101394765)	T	0.119	2368	1.429 (0.317)	6.95 x 10^−6^	0.105	670	1.674 (0.523)	1.43 x 10^−3^	**4.59 x 10**^**−8**^

^a^ Beta values determined based on minor allele

We observed two significant associations for cranial base width: one at 14q21.1 (top SNP rs79272428, p = 1.01 x 10^−8^, **Fig 2A**) and the other at 20q12 (top SNP rs6129564, p = 1.65 x 10^−9^, **Fig 2B**). Notably, the chromosome 20 association exceeded our strict threshold for study-wide statistical significance. For intercanthal width, we observed two significant associations: one at 1p13.3 (top SNP rs619686, p = 4.70 x 10^−8^, **Fig 3A**) and the other at Xq13.2 (top SNP rs11093404, 4.16 x 10^−8^, **Fig 3B**). There were also significant associations with nasal width at 20p11.22 (rs2424399, p = 2.62 x 10^−8^, **Fig 4A**) and nasal ala length at 14q11.2 (top SNP rs8007643, p = 3.36 x 10^−8^, **Fig 4B**). We observed a second independent peak on chromosome 20 for nasal width located 371kb upstream of the main peak. The second peak remained (top SNP rs80186620, p = 5.32x10^-6^, **S21 Fig**) after conditional association analysis adjusting for the effects of rs2424399 on nasal width. Finally we observed a significant association with upper facial depth at 11q22.1 (top SNP rs12786942, p = 4.59 x 10^−8^, **Fig 5**). For all of the above associations, the results were driven primarily by the larger Pittsburgh dataset. The cranial base width (14q21.1), intercanthal width (1p13.3) and upper facial depth associations were at least nominally significant (p < 0.05) in both datasets. Sample-specific association results for all SNPs with p-values less than 5 x 10^−7^ are listed in **S2 Table** for the Pittsburgh sample and **S3 Table** for the Denver sample.

In an attempt to replicate the main findings from the prior two GWA studies in Europeans, we tested previously implicated SNPs against traits from our Pittsburgh dataset that capture similar aspects of morphology. This was not possible for every prior genotype-phenotype association given differences in the measurements available. With that limitation in mind, the Pittsburgh dataset was chosen for comparison because it was the larger of our two datasets and the phenotyping protocol was most similar to prior GWA studies. For Paternoster et al. [17], we attempted to test three of their four genome-wide significant associations, two of which involved nasal ala length (**Table 2**). In our data, nasal ala length showed a nominally significant association (p = 0.018) with rs1982862, an intronic variant in *CACNA2D3*. Conversely, we found no evidence of association between this measure and rs11738462, an intronic variant in *C5orf64*. The previously observed association between *PAX3* and the position of nasion relative to the orbits could not be tested directly. However, we found no evidence of association between the implicated SNP rs7559271 and intercanthal width, which captures aspects of interorbital septum morphology. As a further exploratory analysis we also looked at the association between rs7559271 and several vertical or projective measurements involving nasion, but no significant associations were found for any of these traits. For Liu et al. [18], we attempted to test each of their six previously reported genome-wide significant associations (**Table 2**). We observed a strong association (p = 1.70 x 10^−5^) between nasal ala length and rs4648379, an intronic variant in *PRDM16*. We also observed an association between rs6555969, a SNP near *C5orf50* and upper facial depth (p = 0.005), which is a reasonable approximation of the zygion-nasion distance reported by Liu et al. [18]. To test the association between interorbital distance and rs17447439, an intronic variant in *TP63*, we used measures of intercanthal and outercanthal width; however, we did not observe an association with either measure. Finally, Liu et al. [18] reported associations between SNPs in *PAX3*, *C5orf50* and *COL17A1* and the position of nasion relative to the orbits. We tested these three SNPs in our dataset against intercanthal width, a trait involving roughly similar anatomical components. Notably, we found associations between rs974448 (*PAX3*, p = 0.002) and rs6555969 (*C5orf50*, p = 0.049) and intercanthal width.

**Table 2:** Testing of previously identified genome-wide significant SNPs in European samples.

	Published GWAS Results						Current Results		
Study	Locus	Candidate gene	SNP	Minor allele (MAF)	Beta (p)	Associated phenotype(s)	Closest phenotype(s) in our dataset	Minor allele (MAF)	Beta (p)
Paternoster et al. [17]	12q21.3	*TMTC2*	rs10862567	T (0.31)	0.181 (4.4x10^-8^)	Position of the right endocanthion point	N/A	T (0.32)	N/A
	2q36	*PAX3*	rs7559271	G (0.38)	0.169 (2.2x10^-10^)	Nasion to mid-endocanthion distance	Intercanthal width (Fig 1H)	G (0.37)	0.066 (0.392)
	3p14.3	*CACNA2D3*	rs1982862	A (0.12)	-0.257 (1.8x10^-8^)	Pronasale to alare distance	Nasal ala length (Fig 1N)	A (0.16)	-0.297 (0.018)
	5q12	*C5orf64*	rs11738462	A (0.17)	-0.204 (1.8x10^-8^)	Pronasale to alare distance	Nasal ala length (Fig 1N)	A (0.18)	0.028 (0.818)
Liu et al. [18]	1p36.3	*PRDM16*	rs4648379	T (0.28)	-0.260 (1.1x10^-8^)	Pronasale to alare distance	Nasal ala length (Fig 1N)	T (0.28)	-0.447 (1.7x10^-5^)
	2q36	*PAX3*	rs974448	G (0.17)	0.290 (1.6x10^-8^)	Nasion to orbit distance ^a^	Intercanthal width (Fig 1H)	G (0.17)	0.171 (0.002)
	3q28	*TP63*	rs17447439	G (0.04)	-0.910 (4.4x10^-8^)	Distance between the orbits ^a^	Intercanthal width (Fig 1H)	G (0.04)	0.043 (0.758)
							Outercanthal width (Fig 1I)	G (0.04)	0.261 (0.273)
	5q35.1	*C5orf50*	rs6555969	T (0.33)	0.410 (1.2x10^-9^)	Nasion to zygion distance	Upper facial depth (Fig 1B)	T (0.33)	0.615 (0.005)
					0.260 (2.3x10^-9^)	Nasion to orbit distance ^a^	Intercanthal width (Fig 1H)	T (0.33)	0.159 (0.049)
	10q25.1	*COL17A1*	rs805722	T (0.19)	0.290 (4.0x10^-8^)	Nasion to orbit distance ^a^	Intercanthal width (Fig 1H)	T (0.18)	0.047 (0.628)

^a^ orbit landmark measured from MRI at the approximate location of the pupil

## Discussion

Based on meta-analysis, we observed seven associated loci for five facial traits: cranial base width (**Fig 1A**), intercanthal width (**Fig 1H**), nasal width (**Fig 1K**), nasal ala length (**Fig 1N**), and upper facial depth (**Fig 1B**). The most significant of these, meeting the strict study-wide threshold for significance (i.e., p < 5 x 10^−9^), was the association of cranial base width at 20q12 410kb downstream of *MAFB*, a transcription factor previously implicated in orofacial clefts [29] and facial characteristics in cleft families [30]. However, the *MAFB* SNP associated with clefting was 250kb away and not in LD with the SNP observed here. In addition to orofacial clefting, mutations in *MAFB* cause multicentric carpotarsal osteolysis syndrome, which includes mild facial anomalies. These phenotypes are consistent with the developmental role of *MAFB* in regulating the migration of cranial neural crest cells during the patterning of skeletomuscular features of the head [31]. Altogether, these lines of evidence suggest a possible role for *MAFB* in normal facial variation.

Another association for cranial base width was observed at 14q21.1 in the vicinity of *PAX9*, *SLC25A2*, *MIPOL1*, and *FOXA1*. The homeodomain protein-coding *PAX9* is important for craniofacial development in mice [32,33] and dental development in humans [34]. Using *in situ* hybridization, Peters et al. [32] showed that *Pax9* is expressed throughout the developing cranial base in mice at E13.5. Biological evidence for other genes in this region also suggests possible roles in facial variation including *MIPOL1*, which has been observed to be affected by chromosomal aberrations in patients with craniofacial phenotypes, including holoprosencephaly [35]. Because holoprosencephaly involves alteration in the horizontal spacing of facial structures, variants in genes associated with this condition may also influence measures of craniofacial width in healthy subjects. Taken together, these previous observations point to the plausibility of genetic variants in this region influencing normal facial variation.

Two genetic associations were observed for intercanthal width. One of these was at 1p13.3 within the gene *GSTM2*, which codes an enzyme involved in detoxification of compounds. Among the genes within 250kb of the peak signal are two potentially relevant candidate genes, *GNAI3* and *ALX3*. Mutations in *GNAI3*, which encodes a G protein subunit involved in pharyngeal arch patterning, cause auriculocondylar syndrome, a rare craniofacial disorder [36,37], although hyper- or hypotelorism have not specifically been described. *ALX3* is a homeobox gene essential for head and face development. Mutations in *ALX3* result in frontonasal dysplasia 1 [38] in humans and nasal clefts in mice [39]. Ocular hypertelorism is a prominent feature of frontonasal dysplasia and is believed to result from disruptions of the Hedgehog signaling pathway [40,41]. The second association with intercanthal width was observed for a broad 900kb LD block on Xq13.2. The peak of the diffuse association signal is over *PABP1C1L2A*, which encodes an uncharacterized poly-A binding protein. However, at the edge of the LD block, roughly 500kb centromeric to the peak signal, is *HDAC8*, which encodes a histone deacetylase involved in epigenetic gene silencing during craniofacial development [42]. Mutations in *HDAC8* cause Cornelia de Lange syndrome [43,44], a developmental disorder characterized by facial dysmorphology including hypertelorism. A mutation in *HDAC8* has also been described in a family with an X-linked intellectual disability syndrome with distinctive facial features, which included hypertelorism [45].

A number of other genetic associations with facial traits were observed at loci harboring genes with relevant biological roles. For example, an association with nasal width was observed at 20p11.22 near the *PAX1* gene. Mutations in *PAX1* cause otofaciocervical syndrome [46], characterized by facial dysmorphology, including specific nasal features such as a sunken nasal root and excessive narrowing. *PAX1* plays a role in chondrocyte differentiation [47], which may explain its association with nasal width, a measure of the distance between the left and right cartilaginous nasal alae. Nevertheless, a study of *Pax1* expression in mice showed expression in the pharyngeal arches at E11.5, but not in the developing olfactory placodes [48], so it is unclear how this gene influences nasal development. An association with nasal ala length was observed at 14q11.2 in a region containing an RNase gene cluster plus at least 25 other genes (within about 400kb of the association peak). Among the many genes in region are *ZNF219*, which encodes a transcriptional partner of Sox9 essential for chondrogenesis in mice [49], and *CHD8*, mutations in which are associated with autism spectrum disorder in conjunction with macrocephaly and distinct facial features including a broad nose [50]. A similar story pertains to the association between SNPs on 11q22.1 and upper facial depth. The peak signal occurs within *TRPC6*, which encodes a cation channel subunit mutated in hereditary renal disease [51]. *TRPC6* has no known connection to craniofacial development, but other genes in the region have reported craniofacial expression, including *YAP1* [52].

In aggregate, we observed a number of genetic associations near genes with biologically plausible roles in facial variation. A common theme was that associated loci harbored genes involved in syndromes with craniofacial phenotypes. This result fits with a long-standing hypothesis about the relationship between Mendelian syndromes and complex traits in which common variants near genes causing Mendelian syndromes are involved in related common, complex diseases and traits, including normal phenotypic variation [53]. That being said, for any of the observed associations, it is not clear which variant might be functional, though we hypothesize that the functional variants underlying the statistical signal will be regulatory. Moreover, it is not clear which genes they regulate. Thus, references to implicated genes should always be treated with appropriate caution.

While none of our genome-wide or suggestive (p < 5 x 10^−7^) signals included SNPs implicated in either of the previous two European-focused GWA studies [17,18], we nevertheless found evidence of association when we tested the top SNPs from these studies against comparable phenotypes from our data. Strongest among these was nasal ala length, a lateral projective measure of the nose extending from alar cartilage to the nasal tip, previously associated with 1p36.32 (rs4648379, *PRDM16*) [18] or 3p14.3 (rs1982862, *CACNA2D3* [17]. We found at least nominal associations with both of these regions in our data, with one (rs4648379, *PRDM16*) showing evidence at p = 1.70 x 10^−5^. Both prior GWA studies reported an association between SNPs at 2q35 (*PAX3*) and morphology of the interorbital septum. We tested these SNPs and found an association between rs974448 and intercanthal width (p = 0.002), lending some additional support to the claim that common variants in *PAX3* might influence aspects of normal facial morphology.

Our ability to test previously reported genetic associations was limited by a lack of directly comparable phenotypes, which is related to differences in data collection methods and the type and number of measurements available. In addition, the prior two European GWA studies each used imaging modalities different from the kind used here. Similar factors may also explain some of the discrepancies in association results observed between our two study cohorts. Although care was taken to generate the same set of distance measures in both cohorts, the different 3D cameras and landmarking protocols used could result in different patterns of association. Despite these differences, the measurements from both cohorts were found to be very similar in their overall distributions. Alternative phenotypes, such as multivariate measures of facial shape, can also be used in these types of studies. However, prior attempts to extract shape variation from landmark and surface data in similarly sized samples (e.g., using Procrustes–based methods) have not yielded statistically significant associations [17,18]. One reason for this may be that the effect of any single gene is diluted because the resulting phenotypes represent such a complicated mix of local and global shape features. The problem is highly complex and there is presently little consensus on the most prudent approach to complex facial phenotyping [54]. Fortunately, several promising approaches are on the horizon, such as the BRIM methods being developed by Claes et al. [28]. It is likely that samples an order of magnitude larger than anything available at the moment will be required before we can begin to exploit the richness contained in human 3D facial datasets.

Despite these limitations, we have found evidence of genetic association between chromosomal regions containing genes with important roles in facial development and quantitative traits that characterize key features of the normal human craniofacial complex. In addition to improving our general knowledge of the factors that underlie the diversity of facial forms we see in humans, these genotype-phenotype associations may help us better understand the wide range of phenotypic expression and severity seen in some rare genetic syndromes. Common variants in a number of different genes or regulatory elements may contribute to the expression of dysmorphic phenotypes present in these conditions. Moreover, such associations in healthy individuals may aid the search for clues to the etiology of much more common craniofacial anomalies. For example, three of the traits reported here (cranial base width, nasal width and intercanthal width) have all been previously implicated as potential phenotypic risk factors for orofacial clefting, the most common craniofacial birth defect in humans [55]. Weinberg et al. [15,56] have shown that the unaffected, but genetically at-risk, relatives of cleft-affected individuals exhibit increased breadth through the middle and upper face. The identification of the genes that influence these traits may help us identify important risk-modifiers for clefting [16]. Testing the SNPs implicated here for associations in our cleft families is a future goal of our research group.

## Materials and Methods

### Ethics statement

Institutional ethics (IRB) approval was obtained at each recruitment site and all subjects gave their written informed consent prior to participation (University of Pittsburgh Institutional Review Board #PRO09060553 and #RB0405013; UT Health Committee for the Protection of Human Subjects #HSC-DB-09-0508; Seattle Children’s Institutional Review Board #12107; University of Iowa Human Subjects Office/Institutional Review Board #200912764 and #200710721; Colorado Multiple Institutional Review Board #09–0731; UCSF Human Research Protection Program Committee on Human Research #10–00565).

### Study samples

Our study included two independent samples, each comprised of unrelated self-described White individuals of European ancestry from the United States. The Pittsburgh sample included 2447 unrelated individuals ranging in age from three to 49 years. The majority of these participants (n = 2272) were recruited at research centers in Pittsburgh, Seattle, Houston and Iowa City as part of the FaceBase Consortium’s 3D Facial Norms Dataset, described in detail by Weinberg et al. [57]. The remaining subjects were recruited as healthy controls for a separate study at Pittsburgh on orofacial cleft genetics. The Denver sample included 671 unrelated individuals ranging in age from three to 12 years. These participants were recruited from Denver and San Francisco as part of a separate FaceBase Consortium study of facial shape genetics in multiple ethnic populations [58]. The basic demographic features of these samples are provided in **S4 Table**. In both samples, subjects were excluded if they had a personal history of facial trauma, a personal history of facial reconstructive or plastic surgery, a personal history of orthognathic/jaw surgery or jaw advancement, a personal history of any facial prosthetics or implants, a personal history of any palsy, stroke or neurologic condition affecting the face, a personal or family history of any facial anomaly or birth defect, and/or a personal or family history of any syndrome or congenital condition known to affect the head or face.

### Phenotyping

3D facial surfaces were captured using digital stereophotogrammetry, a standard imaging method resulting in high-density, geometrically accurate point clouds representing the surface contours of the human body [59]. Facial surfaces in the Pittsburgh sample were collected with 3dMD imaging systems (3dMD, Atlanta, GA). Facial surfaces in the Denver sample were imaged using the Creaform Gemini camera system (Quebec, Canada). A common set of 24 standard facial soft-tissue landmarks [60] was collected on each 3D facial surface and the xyz coordinate locations recorded (**S22 Fig**). Landmarks were collected manually in the Pittsburgh sample as described in Weinberg et al. [57]. An automated landmark collection method was used in the Denver sample. From these landmarks, we calculated 20 linear distances that correspond to craniofacial measurements commonly used in clinical assessment [61]. These measurements are shown in **Fig 1** and listed in **S5 Table**. For bilateral measurements, values were summed across the left and right sides in order to minimize the number of traits tested. Trait values were inspected for outliers by computing age- and sex-specific z-scores.

### Genotyping, quality checks, imputation, and population structure

For each study sample, genotyping was performed by the Center for Inherited Disease Research (CIDR). Saliva samples were used to genotype 3,186 participants for 964,193 SNPs on the Illumina (San Diego, CA) OmniExpress+Exome v1.2 array plus 4,322 custom SNPs chosen in regions of interest based on previous studies of the genetics of facial variation. In addition, 70 duplicates and 72 HapMap control samples were genotyped for quality assurance purposes. Data cleaning was performed by the University of Washington Genetics Coordinating Center (UWGCC) using standard analysis pipelines implemented in the R Environment for Statistical Computing, as previously described [62]. These analyses include interrogating samples for genetic sex, chromosomal anomalies, relatedness among participants, missing call rate, and batch effects, and interrogating SNPs for missing call rate, discordance between duplicate samples, Mendelian errors (as measured in HapMap control parent-offspring trios), Hardy-Weinberg equilibrium, and differences in allele frequency and heterozygosity between sexes (for autosomal and pseudo-autosomal SNPs). Supplemental **S6 Table** shows the number of SNPs omitted and retained for each quality filter.

Imputation was performed to capture information on unobserved SNPs as well as sporadically missing genotypes among genotyped SNPs, using haplotypes from the 1000 Genomes Project [63] Phase 3 reference panel of 2,504 samples from 26 worldwide populations. First, pre-phasing was performed in SHAPEIT2 [64], and then imputation of 34,985,077 variants was performed in IMPUTE2 [65,66]. Masked variant analysis–that is, imputation of genotyped SNPs as though they were unobserved in order to assess imputation quality–showed high concordance between imputed and observed genotypes (0.998 for SNPs with MAF < 0.05 and 0.982 for SNPs with MAF ≥ 0.05) indicating high quality imputation. Imputed SNPs were included in analyses if the minimum genotype probability for a given variant was greater than 50%.

Principal component analysis using 96,700 autosomal SNPs pruned from the total panel based on call rate (> 95%), MAF (> 0.05), and LD (pairwise r^2^ < 0.1 in a sliding window of 10 Mb), was used to assess population structure. Supplemental **S23 Fig** depicts the observed genetic structure of the population across the first two principal components of ancestry (i.e., eigenvectors from the PCA). Linear regression was used to test the association between each PC, as the dependent variable, and each SNP in the genome. These analyses confirmed that none of the first 20 principal components were due to local variation in specific genomic regions.

### Statistical approach

Prior to genetic analysis, each of the 20 linear distance measures was adjusted for the effects of sex, age, age^2^, height, weight, and facial size (calculated as the geometric mean of the linear distance measures) using linear regression in order to generate 20 adjusted phenotypes (i.e., residuals). The inclusion of age and age^2^ as covariates was done in an effort to adjust for both linear and non-linear aspects of age on the phenotypes. After model fitting different sets of covariates, including more complicated spline functions, we settled on a combination of age and age^2^ as the most reasonable approach based on akaike information criterion values calculated across age-adjustment models. Linear models were then used to test genetic association between each phenotype and each SNP, under the additive genetic model, while simultaneously adjusting for the first four principal components of ancestry. For SNPs on the X chromosome we coded hemizygous males as 0/2 so they are on the same scale as 0/1/2 females. Analyses were performed separately for the Pittsburgh and Denver cohorts, and combined via inverse variance-weighted meta-analysis. To appropriately model SNP effects, we required that the minor allele be present in at least 30 participants, corresponding to a MAF threshold of 0.6% in the Pittsburgh cohort and 2% in the Denver cohort. SNPs meeting the minor allele count criterion in both Pittsburgh and Denver cohorts were included in the meta-analysis. The final number of genotyped SNPs available for analysis after minor allele filtering was 659,955 for the Pittsburgh sample, 638,772 for the Denver sample, and 637,391 for the meta-analysis. The number of imputed and total (genotyped plus imputed) SNPs is available in **S7 Table**.

Given the large number of tests, we used the conventional threshold of p < 5 x 10^−8^ (i.e., Bonferroni correction for 1 million tests) for genome-wide statistical significance. Because we expect many of our traits to be correlated, we used the eigenvalue method described by Li and Ji [67] to determine that the effective number of independent traits was 10. Thus, we set the threshold for study-wide statistical significance at p < 5 x 10^−9^ (i.e. p < 5 x 10^−8^ divided by 10). Because these thresholds are very conservative, we also reported “suggestive” evidence of association of p < 5 x 10^−7^ in **S1–S3 Tables**. Phenotypes were generated using the R Environment for Statistical Computing, and genetic association was performed using PLINK [68].

### Availability of data

All of the phenotypic measures and genotypic markers used here are available to the research community through the dbGaP controlled access repository (http://www.ncbi.nlm.nih.gov/gap) at accession number: phs000949.v1.p1. The raw source data for the phenotypes–the 3D facial surface models–are available for the 3D Facial Norms dataset through the FaceBase Consortium (www.facebase.org). Finally, searchable results datasets of the p-values from the studies reported here are available through the FaceBase Human Genomics Analysis Interface (facebase.sdmgenetics.pitt.edu).

## Supporting Information

S1 Table Associations with p-values < 5 x 10^−7^ for all 20 traits from genome-wide meta-analysis. (XLSX)

S2 Table Associations with p-values < 5 x 10^−7^ for all 20 traits from genome-wide analysis of the Pittsburgh sample. (XLSX)

S3 Table Associations with p-values < 5 x 10^−7^ for all 20 traits from genome-wide analysis of the Denver sample. (XLSX)

S4 Table Descriptive statistics of the two study samples. (DOCX)

S5 Table List of linear distance measurements. (DOCX)

S6 Table Number of SNPs omitted and retained for each quality filter. (DOCX)

S7 Table Final number of SNPs used in our analysis. (DOCX)

S1 Fig Manhattan plots for cranial base width. (A) meta-analysis results, (B) Pittsburgh sample results, and (C) Denver sample results. Lines for p-value thresholds set at 5 x 10^−8^ for genome-wide significance and 5 x 10^−7^ for suggestive significance.
(PDF)

S2 Fig Manhattan plots for upper facial depth. (A) meta-analysis results, (B) Pittsburgh sample results, and (C) Denver sample results. Lines for p-value thresholds set at 5 x 10^−8^ for genome-wide significance and 5 x 10^−7^ for suggestive significance.
(PDF)

S3 Fig Manhattan plots for middle facial depth. (A) meta-analysis results, (B) Pittsburgh sample results, and (C) Denver sample results. Lines for p-value thresholds set at 5 x 10^−8^ for genome-wide significance and 5 x 10^−7^ for suggestive significance.
(PDF)

S4 Fig Manhattan plots for lower facial depth. (A) meta-analysis results, (B) Pittsburgh sample results, and (C) Denver sample results. Lines for p-value thresholds set at 5 x 10^−8^ for genome-wide significance and 5 x 10^−7^ for suggestive significance.
(PDF)

S5 Fig Manhattan plots for morphological facial height. (A) meta-analysis results, (B) Pittsburgh sample results, and (C) Denver sample results. Lines for p-value thresholds set at 5 x 10^−8^ for genome-wide significance and 5 x 10^−7^ for suggestive significance.
(PDF)

S6 Fig Manhattan plots for upper facial height. (A) meta-analysis results, (B) Pittsburgh sample results, and (C) Denver sample results. Lines for p-value thresholds set at 5 x 10^−8^ for genome-wide significance and 5 x 10^−7^ for suggestive significance.
(PDF)

S7 Fig Manhattan plots for lower facial height. (A) meta-analysis results, (B) Pittsburgh sample results, and (C) Denver sample results. Lines for p-value thresholds set at 5 x 10^−8^ for genome-wide significance and 5 x 10^−7^ for suggestive significance.
(PDF)

S8 Fig Manhattan plots for intercanthal width. (A) meta-analysis results, (B) Pittsburgh sample results, and (C) Denver sample results. Lines for p-value thresholds set at 5 x 10^−8^ for genome-wide significance and 5 x 10^−7^ for suggestive significance.
(PDF)

S9 Fig Manhattan plots for outercanthal width. (A) meta-analysis results, (B) Pittsburgh sample results, and (C) Denver sample results. Lines for p-value thresholds set at 5 x 10^−8^ for genome-wide significance and 5 x 10^−7^ for suggestive significance.
(PDF)

S10 Fig Manhattan plots for palpebral fissure width. (A) meta-analysis results, (B) Pittsburgh sample results, and (C) Denver sample results. Lines for p-value thresholds set at 5 x 10^−8^ for genome-wide significance and 5 x 10^−7^ for suggestive significance.
(PDF)

S11 Fig Manhattan plots for nasal width. (A) meta-analysis results, (B) Pittsburgh sample results, and (C) Denver sample results. Lines for p-value thresholds set at 5 x 10^−8^ for genome-wide significance and 5 x 10^−7^ for suggestive significance.
(PDF)

S12 Fig Manhattan plots for subnasal width. (A) meta-analysis results, (B) Pittsburgh sample results, and (C) Denver sample results. Lines for p-value thresholds set at 5 x 10^−8^ for genome-wide significance and 5 x 10^−7^ for suggestive significance.
(PDF)

S13 Fig Manhattan plots for nasal protrusion. (A) meta-analysis results, (B) Pittsburgh sample results, and (C) Denver sample results. Lines for p-value thresholds set at 5 x 10^−8^ for genome-wide significance and 5 x 10^−7^ for suggestive significance.
(PDF)

S14 Fig Manhattan plots for nasal ala length. (A) meta-analysis results, (B) Pittsburgh sample results, and (C) Denver sample results. Lines for p-value thresholds set at 5 x 10^−8^ for genome-wide significance and 5 x 10^−7^ for suggestive significance.
(PDF)

S15 Fig Manhattan plots for nasal height. (A) meta-analysis results, (B) Pittsburgh sample results, and (C) Denver sample results. Lines for p-value thresholds set at 5 x 10^−8^ for genome-wide significance and 5 x 10^−7^ for suggestive significance.
(PDF)

S16 Fig Manhattan plots for nasal bridge length. (A) meta-analysis results, (B) Pittsburgh sample results, and (C) Denver sample results. Lines for p-value thresholds set at 5 x 10^−8^ for genome-wide significance and 5 x 10^−7^ for suggestive significance.
(PDF)

S17 Fig Manhattan plots for labial fissure width. (A) meta-analysis results, (B) Pittsburgh sample results, and (C) Denver sample results. Lines for p-value thresholds set at 5 x 10^−8^ for genome-wide significance and 5 x 10^−7^ for suggestive significance.
(PDF)

S18 Fig Manhattan plots for philtrum length. (A) meta-analysis results, (B) Pittsburgh sample results, and (C) Denver sample results. Lines for p-value thresholds set at 5 x 10^−8^ for genome-wide significance and 5 x 10^−7^ for suggestive significance.
(PDF)

S19 Fig Manhattan plots for upper lip height. (A) meta-analysis results, (B) Pittsburgh sample results, and (C) Denver sample results. Lines for p-value thresholds set at 5 x 10^−8^ for genome-wide significance and 5 x 10^−7^ for suggestive significance.
(PDF)

S20 Fig Manhattan plots for lower lip height. (A) meta-analysis results, (B) Pittsburgh sample results, and (C) Denver sample results. Lines for p-value thresholds set at 5 x 10^−8^ for genome-wide significance and 5 x 10^−7^ for suggestive significance.
(PDF)

S21 Fig **LocusZoom plots comparing the (A) discovery analysis, and (B) conditional analysis for the observed genetic association of nasal width near PAX1 at 20p11.22**. Genetic association (left y-axis; log10-transformed p-values) is shown for genotyped SNPs depicted as stars and imputed SNPs depicted as circles. Shading of the points represent the linkage disequilibrium (r^2^) between each SNP and the rs2424399 (the top SNP from the discovery analysis), indicated by purple shading. The blue overlay shows the recombination rate (right y-axis). Positions of genes are shown below the plot. In the discovery analysis, a possible second peak in low-LD with the rs2424399 was observed around chromosomal position 22.0 Mb. After conditioning on rs2424399, variants at position 22.0 Mb showed some independent evidence of association, although not meeting genome-wide or suggestive thresholds for significance.
(PDF)

S22 Fig 3D facial surface model showing the location of the 24 standard landmarks used to generate the linear distances. Landmarks shown in frontal view (A) are n = nasion; prn = pronasale; sn = subnasale; ls = labiale superius; sto = stomion; li = labiale inferius; sl = sublabiale; gn = gnathion; en = endocanthion; ex = exocanthion; al = alare; sbal = subalare; cph = crista philtra; ch = chelion (for bilateral points only right side labeled). Landmarks shown in the lateral view (B) are ac = alar curvature point and t = tragion (only left landmark shown for these two bilateral points).
(PDF)

S23 Fig Plot showing population stratification across the first two principal components of ancestry (EV1 and EV2). The proportion of total genetic variation explained by each principal component of ancestry is indicated on the axis.
(PDF)

## References

[1] Byard PJ, Poosha DVR, Satyanarayana M, Rao DC. Family resemblance for components of craniofacial size and shape. J Craniofac Genet Dev Biol. 1985;5:229–238. 4044786

[2] Carels C, Van Cauwenberghe N, Savoye I, Willems G, Loos R, Derom C, et al A quantitative genetic study of cephalometric variables in twins. Orthod Craniofacial Res. 2001;4:130–140. 10.1034/j.1600-0544.2001.040303.x 11553097

[3] Ermakov S, Kobyliansky E, Livshits G. Complex segregation analysis of two principal components derived from horizontal and vertical head size traits. Ann Hum Biol. 2006;33:546–556. 17381053 10.1080/03014460600931046

[4] Martínez-Abadías N, Esparza M, Sjøvold T, González-José R, Santos M, Hernández M. Heritability of human cranial dimensions: comparing the evolvability of different cranial regions. J Anat. 2009;214:19–35. 10.1111/j.1469-7580.2008.01015.x 19166470 PMC2667914

[5] Šešelj M, Duren DL, Sherwood RJ. Heritability of the human craniofacial complex. Anat Rec. 2015;298:1535–1547. 10.1002/ar.23186 PMC6407862 26097051

[6] Hennekam RC, Krantz ID, Allanson JE. Gorlin's Syndromes of the Head and Neck. Fifth Edition Oxford: Oxford University Press; 2010.

[7] Morriss-Kay GM. Craniofacial defects in AP-2 null mutant mice. Bioessays. 1996;18:785–788. 8885715 10.1002/bies.950181004

[8] Hallgrimsson B, Brown JJY, Ford-Hutchinson AF, Sheets HD, Zelditch ML, Jirik FR. The brachymorph mouse and the developmental-genetic basis for canalization and morphological integration. Evol Dev. 2006;8:61–73. 16409383 10.1111/j.1525-142X.2006.05075.x

[9] Perlyn CA, DeLeon VB, Babbs C, Govier D, Burell L, Darvann T, et al The craniofacial phenotype of the Crouzon mouse: analysis of a model for syndromic craniosynostosis using three-dimensional microCT. Cleft Palate Craniofac J. 2006;43:740–747. 17105336 10.1597/05-212

[10] Haworth K, Breen M, Binns M, Hopkinson DA, Edwards YH. The canine homeobox gene MSX2: sequence, chromosome assignment and genetic analysis in dogs of different breeds. Anim Genet. 2001;32(1):32–6. 11419342 10.1046/j.1365-2052.2001.00702.x

[11] Klingenberg CP, Leamy LJ, Routman EJ, Cheverud JM. Genetic architecture of mandible shape in mice: effects of quantitative trait loci analyzed by geometric morphometrics. Genetics. 2001;157:785–802. 11156997 10.1093/genetics/157.2.785 PMC1461535

[12] Sherwood RJ, Duren DL, Havill LM, Rogers J, Cox LA, Towne B, et al A genomewide linkage scan for quantitative trait loci influencing the craniofacial complex in baboons (Papio hamadryas spp.). Genetics. 2008;180:619–628. 10.1534/genetics.108.090407 18757921 PMC2535711

[13] Maga AM, Navarro N, Cunningham ML, Cox TC. Quantitative trait loci affecting the 3D skull shape and size in mouse and prioritization of candidate genes in-silico. Front Physiol. 2015;6:92 10.3389/fphys.2015.00092 25859222 PMC4374467

[14] Pallares LF, Carbonetto P, Gopalakrishnan S, Parker CC, Ackert-Bicknell CL, Palmer AA, et al Mapping of craniofacial traits in outbred mice identifies major developmental genes involved in shape determination. PLoS Genet. 2015;11:e1005607 10.1371/journal.pgen.1005607 26523602 PMC4629907

[15] Weinberg SM, Naidoo S, Bardi KM, Brandon CA, Neiswanger K, Resick JM, et al Face shape of unaffected parents with cleft affected offspring: combining three-dimensional surface imaging and geometric morphometrics. Orthod Craniofacial Res. 2009;12:271–281. 10.1111/j.1601-6343.2009.01462.x PMC2765674 19840279

[16] Boehringer S, van der Lijn F, Liu F, Günther M, Sinigerova S, Nowak S, et al Genetic determination of human facial morphology: links between cleft-lips and normal variation. Eur J Hum Genet. 2011;19:1192–1197. 10.1038/ejhg.2011.110 21694738 PMC3198142

[17] Paternoster L, Zhurov AI, Toma AM, Kemp JP, St Pourcain B, Timpson NJ, et al Genome-wide association study of three-dimensional facial morphology identifies a variant in PAX3 associated with nasion position. Am J Hum Genet. 2012;90:478–485. 10.1016/j.ajhg.2011.12.021 22341974 PMC3309180

[18] Liu F, van der Lijn F, Schurmann C, Zhu G, Chakravarty MM, Hysi PG, et al A genome-wide association study identifies five loci influencing facial morphology in Europeans. PLoS Genet. 2012;8:e1002932 10.1371/journal.pgen.1002932 23028347 PMC3441666

[19] Yamaguchi T, Maki K, Shibasaki Y. Growth hormone receptor gene variant and mandibular height in the normal Japanese population. Am J Orthod Dentofacial Orthop. 2001;119:650–653. 11395710 10.1067/mod.2001.114536

[20] Coussens AK, van Daal A. Linkage disequilibrium analysis identifies an FGFR1 haplotype-tag SNP associated with normal variation in craniofacial shape. Genomics. 2005;85:563–73. 15820308 10.1016/j.ygeno.2005.02.002

[21] Kang EH, Yamaguchi T, Tajima A, Nakajima T, Tomoyasu Y, Watanabe M, et al Association of the growth hormone receptor gene polymorphisms with mandibular height in a Korean population. Arch Oral Biol. 2009;54:556–562. 10.1016/j.archoralbio.2009.03.002 19344888

[22] Sasaki Y, Satoh K, Hayasaki H, Fukumoto S, Fujiwara T, Nonaka K. The P561T polymorphism of the growth hormone receptor gene has an inhibitory effect on mandibular growth in young children. Eur J Orthod. 2009;31:536–541. 10.1093/ejo/cjp017 19447840

[23] Tomoyasu Y, Yamaguchi T, Tajima A, Nakajima T, Inoue I, Maki K. Further evidence for an association between mandibular height and the growth hormone receptor gene in a Japanese population. Am J Orthod Dentofacial Orthop. 2009;136:536–541. 10.1016/j.ajodo.2007.10.054 19815155

[24] Ermakov S, Rosenbaum MG, Malkin I, Livshits G. Family-based study of association between ENPP1 genetic variants and craniofacial morphology. Ann Hum Biol. 2010;37:754–766. 10.3109/03014461003639231 20446819

[25] Gómez-Valdés JA, Hünemeier T, Contini V, Acuña-Alonzo V, Macin G, Ballesteros-Romero M, et al Fibroblast growth factor receptor 1 (FGFR1) variants and craniofacial variation in Amerindians and related populations. Am J Hum Biol. 2013;25:12–19. 10.1002/ajhb.22331 23070782

[26] Peng S, Tan J, Hu S, Zhou H, Guo J, Jin L, et al Detecting genetic association of common human facial morphological variation using high density 3D image registration. PLoS Comp Biol. 2013;9:e1003375. 10.1371/journal.pcbi.1003375 PMC3854494 24339768

[27] Bayram S, Basciftci FA, Kurar E. Relationship between P561T and C422F polymorphisms in growth hormone receptor gene and mandibular prognathism. Angle Orthodont. 2014;84(5):803–9. 10.2319/091713-680.1 24654940 PMC8641272

[28] Claes P, Liberton DK, Daniels K, Rosana KM, Quillen EE, Pearson LN, et al Modeling 3D facial shape from DNA. PLoS Genet. 2014;10:e1004224 10.1371/journal.pgen.1004224 24651127 PMC3961191

[29] Beaty TH, Murray JC, Marazita ML, Munger RG, Ruczinski I, Hetmanski JB, et al A genome-wide association study of cleft lip with and without cleft palate identifies risk variants near MAFB and ABCA4. Nat Genet. 2010;42:525–529. 10.1038/ng.580 20436469 PMC2941216

[30] Miller SF, Weinberg SM, Nidey N, Defay D, Marazita ML, Wehby G, et al Exploratory genotype-phenotype correlations of facial form and asymmetry in unaffected relatives of children with non-syndromic cleft lip and/or palate. J Anat. 2014;224:688–709. 10.1111/joa.12182 24738728 PMC4025896

[31] McGonnell IM, McKay IJ, Graham A. A population of caudally migrating cranial neural crest cells: functional and evolutionary implications. Dev Biol. 2001;236:354–363. 11476577 10.1006/dbio.2001.0330

[32] Peters H, Neubuser A, Kratochwil K, Balling R. Pax9-deficient mice lack pharyngeal pouch derivatives and teeth and exhibit craniofacial and limb abnormalities. Genes Dev. 1998;12:2735–2747. 9732271 10.1101/gad.12.17.2735 PMC317134

[33] Sasaki Y, O'Kane S, Dixon J, Dixon MJ, Ferguson MW. Temporal and spatial expression of Pax9 and Sonic hedgehog during development of normal mouse palates and cleft palates in TGF-beta3 null embryos. Arch Oral Biol. 2007;52:260–267. 17097601 10.1016/j.archoralbio.2006.09.012

[34] Cooper LF. Treatment of nonsyndromic anomalies of tooth number In: Wright J, editor. Craniofacial and Dental Developmental Defects: Diagnosis and Management. Switzerland: Springer International Publishing; 2015 p. 49–61.

[35] Kamnasaran D, O'Brien PC, Zackai EH, Muenke M, Ferguson-Smith MA, Cox DW. Rearrangement in the PITX2 and MIPOL1 genes in a patient with a t(4;14) chromosome. Eur J Hum Genet. 2003;11:315–324. 12700605 10.1038/sj.ejhg.5200963

[36] Rieder MJ, Green GE, Park SS, Stamper BD, Gordon CT, Johnson JM, et al A human homeotic transformation resulting from mutations in PLCB4 and GNAI3 causes auriculocondylar syndrome. Am J Hum Genet. 2012;90:907–914. 10.1016/j.ajhg.2012.04.002 22560091 PMC3376493

[37] Romanelli Tavares VL, Gordon CT, Zechi-Ceide RM, Kokitsu-Nakata NM, Voisin N, Tan TY, et al Novel variants in GNAI3 associated with auriculocondylar syndrome strengthen a common dominant negative effect. Eur J Hum Genet. 2015;23:481–485. 10.1038/ejhg.2014.132 25026904 PMC4666574

[38] Twigg SR, Versnel SL, Nürnberg G, Lees MM, Bhat M, Hammond P, et al Frontorhiny, a distinctive presentation of frontonasal dysplasia caused by recessive mutations in the ALX3 homeobox gene. Am J Hum Genet. 2009;84:698–705. 10.1016/j.ajhg.2009.04.009 19409524 PMC2681074

[39] Beverdam A, Brouwer A, Reijnen M, Korving J, Meijlink F. Severe nasal clefting and abnormal embryonic apoptosis in Alx3/Alx4 double mutant mice. Development. 2001;128:3975–3986. 11641221 10.1242/dev.128.20.3975

[40] Brugmann SA, Allen NC, James AW, Mekonnen Z, Madan E, Helms JA. A primary cilia-dependent etiology for midline facial disorders. Hum Mol Genet. 2010;19:1577–1592. 10.1093/hmg/ddq030 20106874 PMC2846163

[41] Young NM, Chong HJ, Hu D, Hallgrimsson B, Marcucio RS. Quantitative analyses link modulation of sonic hedgehog signaling to continuous variation in facial growth and shape. Development. 2010;137:3405–3409. 10.1242/dev.052340 20826528 PMC2947484

[42] Haberland M, Mokalled MH, Montgomery RL, Olson EN. Epigenetic control of skull morphogenesis by histone deacetylase 8. Genes Dev. 2009;23(14):1625–30. 10.1101/gad.1809209 19605684 PMC2714711

[43] Deardorff MA, Bando M, Nakato R, Watrin E, Itoh T, Minamino M, et al HDAC8 mutations in Cornelia de Lange syndrome affect the cohesin acetylation cycle. Nature. 2012;489:313–317. 10.1038/nature11316 22885700 PMC3443318

[44] Parenti I, Gervasini C, Pozojevic J, Wendt KS, Watrin E, Azzollini J, et al Expanding the clinical spectrum of the "HDAC8-phenotype"—Implications for molecular diagnostics, counselling and risk prediction. Clin Genet. 2015;in press. 10.1111/cge.12717 26671848

[45] Harakalova M, van den Boogaard MJ, Sinke R, van Lieshout S, van Tuil MC, Duran K, et al X-exome sequencing identifies a HDAC8 variant in a large pedigree with X-linked intellectual disability, truncal obesity, gynaecomastia, hypogonadism and unusual face. J Med Genet. 2012;49:539–543. 10.1136/jmedgenet-2012-100921 22889856

[46] Pohl E, Aykut A, Beleggia F, Karaca E, Durmaz B, Keupp K, et al A hypofunctional PAX1 mutation causes autosomal recessively inherited otofaciocervical syndrome. Hum Genet. 2013;132:1311–1320. 10.1007/s00439-013-1337-9 23851939

[47] Takimoto A, Mohri H, Kokubu C, Hiraki Y, Shukunami C. Pax1 acts as a negative regulator of chondrocyte maturation. Exp Cell Res. 2013;319:3128–3139. 10.1016/j.yexcr.2013.09.015 24080012

[48] Shimizu H, Kubo A, Uchibe K, Hashimoto M, Yokoyama S, Takada S, et al The AERO system: a 3D-like approach for recording gene expression patterns in the whole mouse embryo. PLoS One. 2013;8:e75754 10.1371/journal.pone.0075754 24146773 PMC3797748

[49] Takigawa Y, Hata K, Muramatsu S, Amano K, Ono K, Wakabayashi M, et al The transcription factor Znf219 regulates chondrocyte differentiation by assembling a transcription factory with Sox9. J Cell Sci. 2010;123:3780–3788. 10.1242/jcs.071373 20940257

[50] Bernier R, Golzio C, Xiong B, Stessman H, Coe BP, Penn O, et al Disruptive CHD8 mutations define a subtype of autism early in development. Cell. 2014;158:263–276. 10.1016/j.cell.2014.06.017 24998929 PMC4136921

[51] Winn MP, Conlon PJ, Lynn KL, Farrington MK, Creazzo T, Hawkins AF, et al A mutation in the TRPC6 cation channel causes familial focal segmental glomerulosclerosis. Science. 2005;308:1801–1804. 15879175 10.1126/science.1106215

[52] Williamson KA, Rainger J, Floyd JA, Ansari M, Meynert A, Aldridge KV, et al Heterozygous loss-of-function mutations in YAP1 cause both isolated and syndromic optic fissure closure defects. Am J Hum Genet. 2014;94:295–302. 10.1016/j.ajhg.2014.01.001 24462371 PMC3928658

[53] Hirschhorn JN, Daly MJ. Genome-wide association studies for common diseases and complex traits. Nat Rev Genet. 2005;6:95–108. 15716906 10.1038/nrg1521

[54] Hallgrimsson B, Mio W, Marcucio RS, Spritz R. Let’s face it—complex traits are just not that simple. PLoS Genet. 2014;10:e1004724 10.1371/journal.pgen.1004724 25375250 PMC4222688

[55] Leslie EJ, Marazita ML. Genetics of cleft lip and cleft palate. Am J Med Genet Part C Semin Med Genet. 2013;163:246–258. 10.1002/ajmg.c.31381 PMC3925974 24124047

[56] Weinberg SM, Neiswanger K, Richtsmeier JT, Maher BS, Mooney MP, Siegel MI, et al Three-dimensional morphometric analysis of craniofacial shape in the unaffected relatives of individuals with nonsyndromic orofacial clefts: a possible marker for genetic susceptibility. Am J Med Genet Part A. 2008;146A:409–420. 10.1002/ajmg.a.32177 18203157

[57] Weinberg SM, Raffensperger ZD, Kesterke MJ, Heike CL, Cunningham ML, Hecht JT, et al The 3D Facial Norms Database: Part 1. A web-based craniofacial anthropometric and image repository for the clinical and research community. Cleft Palate Craniofac J. 2015;in press. 10.1597/15-199 PMC4841760 26492185

[58] Hochheiser H, Aronow BJ, Artinger K, Beaty TH, Brinkley JF, Chai Y, et al The FaceBase Consortium: A comprehensive program to facilitate craniofacial research. Dev Biol. 2011;355:175–182. 10.1016/j.ydbio.2011.02.033 21458441 PMC3440302

[59] Lane C, Harrell W. Completing the 3-dimensional picture. Am J Orthod Dentofacial Orthop. 2008;133:612–620. 10.1016/j.ajodo.2007.03.023 18405826

[60] Kolar JC, Salter EM. Craniofacial Anthropometry: Practical Measurement of the Head and Face for Clinical, Surgical and Research Use. Springfield: Charles C. Thomas; 1997.

[61] Farkas LG. Anthropometry of the Head and Face. Second Edition New York: Raven Press; 1994.

[62] Laurie CC, Doheny KF, Mirel DB, Pugh EW, Bierut LJ, Bhangale T, et al Quality control and quality assurance in genotypic data for genome-wide association studies. Genet Epidemiol. 2010;34:591–602. 10.1002/gepi.20516 20718045 PMC3061487

[63] 1000 Genomes Project Consortium, Abecasis GR, Auton A, Brooks LD, DePristo MA, Durbin RM, et al An integrated map of genetic variation from 1,092 human genomes. Nature. 2012;491:56–65. 10.1038/nature11632 23128226 PMC3498066

[64] Delaneau O, Zagury JF, Marchini J. Improved whole-chromosome phasing for disease and population genetic studies. Nat Methods. 2013;10:5–6. 10.1038/nmeth.2307 23269371

[65] Howie B, Donnelly P, Marchini J. A flexible and accurate genotype imputation method for the next generation of genome-wide association studies. PLoS Genet. 2009;5:e1000529 10.1371/journal.pgen.1000529 19543373 PMC2689936

[66] Howie B, Marchini J, Stephens M. Genotype imputation with thousands of genomes. G3. 2011;1:457–470. 10.1534/g3.111.001198 22384356 PMC3276165

[67] Li J, Ji L. Adjusting multiple testing in multilocus analyses using the eigenvalues of a correlation matrix. Heredity. 2005;95:221–227. 16077740 10.1038/sj.hdy.6800717

[68] Purcell S, Neale B, Todd-Brown K, Thomas L, Ferreira MA, Bender D, et al PLINK: a tool set for whole-genome association and population-based linkage analyses. Am J Hum Genet. 2007;81:559–575. 17701901 10.1086/519795 PMC1950838

