# Supplementary material for: Genome-Wide Association Study Reveals Multiple Loci Influencing Normal Human Facial Morphology
Source: PLoS Genet. 2016 Aug 25;12(8):e1006149. doi: 10.1371/journal.pgen.1006149 (PMC4999139; doi:10.1371/journal.pgen.1006149)

S4 Table. Descriptive statistics for the two study samples

|  | **Pittsburgh Sample (N = 2447)** | | | | | | **Denver Sample (N = 671)** | | | | | |
| --- | --- | --- | --- | --- | --- | --- | --- | --- | --- | --- | --- | --- |
|  | **Male** | | **Female** | | **Total** | | **Male** | | **Female** | | **Total** | |
|  | **N** | **Mean (se)** | **N** | **Mean (se)** | **N** | **Mean (se)** | **N** | **Mean (se)** | **N** | **Mean (se)** | **N** | **Mean (se)** |
| Age(yr) | 939 | 21.22 (0.31) | 1508 | 23.64 (0.23) | 2447 | 22.71 (0.19) | 347 | 7.77 (0.14) | 324 | 7.56 (0.15) | 671 | 7.66 (0.10) |
| Height(cm) | 939 | 168.20 (0.81) | 1508 | 160.47 (0.42) | 2447 | 163.4 (0.41) | 347 | 129.95 (0.86) | 324 | 128.26 (1.02) | 671 | 129.14 (0.66) |
| Weight(kg) | 939 | 69.61 (0.87) | 1508 | 61.77 (0.49) | 2447 | 64.78 (0.46) | 347 | 30.31 (0.63) | 324 | 30.13 (0.68) | 671 | 30.22 (0.46) |
| Cranial base width (mm) | 921 | 144.47 (0.29) | 1462 | 137.60 (0.17) | 2383 | 140.25 (0.17) | 347 | 135.50 (0.39) | 324 | 131.34 (0.43) | 671 | 133.49 (0.30) |
| Upper facial depth (mm) ^a^ | 921 | 245.59 (0.57) | 1462 | 234.44 (0.31) | 2383 | 238.75 (0.31) | 347 | 219.48 (0.67) | 323 | 213.92 (0.68) | 670 | 216.80 (0.49) |
| Middle facial depth (mm) ^a^ | 921 | 256.06 (0.66) | 1462 | 243.47 (0.35) | 2383 | 248.34 (0.35) | 347 | 227.46 (0.77) | 324 | 221.24 (0.77) | 671 | 224.46 (0.56) |
| Lower facial depth (mm) ^a^ | 891 | 288.78 (0.94) | 1440 | 272.90 (0.48) | 2331 | 278.97 (0.49) | 347 | 251.36 (0.93) | 324 | 243.77 (0.94) | 671 | 247.70 (0.68) |
| Morphological facial height (mm) | 906 | 121.46 (0.40) | 1483 | 116.10 (0.22) | 2389 | 118.13 (0.21) | 347 | 98.54 (0.39) | 324 | 95.99 (0.43) | 671 | 97.31 (0.29) |
| Upper facial height (mm) | 938 | 74.89 (0.24) | 1506 | 72.57 (0.15) | 2444 | 73.46 (0.13) | 347 | 60.43 (0.25) | 324 | 58.93 (0.28) | 671 | 59.71 (0.19) |
| Lower facial height (mm) | 906 | 69.93 (0.25) | 1483 | 65.31 (0.15) | 2389 | 67.06 (0.14) | 347 | 59.43 (0.24) | 324 | 57.59 (0.26) | 671 | 58.54 (0.18) |
| Intercanthal width (mm) | 937 | 32.17 (0.10) | 1504 | 31.16 (0.07) | 2441 | 31.54 (0.06) | 347 | 32.95 (0.11) | 324 | 32.27 (0.12) | 671 | 32.62 (0.08) |
| Outercanthal width (mm) | 918 | 86.86 (0.19) | 1484 | 84.57 (0.12) | 2402 | 85.45 (0.11) | 347 | 91.84 (0.26) | 323 | 89.75 (0.27) | 670 | 90.83 (0.19) |
| Palpebral fissure width (mm) ^a^ | 917 | 56.08 (0.15) | 1482 | 54.93 (0.11) | 2399 | 55.37 (0.09) | 347 | 61.99 (0.19) | 323 | 60.51 (0.20) | 670 | 61.27 (0.14) |
| Nasal width (mm) | 937 | 34.58 (0.12) | 1507 | 32.12 (0.07) | 2444 | 33.06 (0.07) | 347 | 29.91 (0.12) | 324 | 29.16 (0.13) | 671 | 29.55 (0.09) |
| Subnasal width (mm) | 936 | 18.66 (0.09) | 1506 | 17.14 (0.06) | 2442 | 17.72 (0.05) | 347 | 18.40 (0.07) | 324 | 17.76 (0.08) | 671 | 18.09 (0.05) |
| Nasal protrusion (mm) | 939 | 19.81 (0.10) | 1508 | 19.52 (0.06) | 2447 | 19.63 (0.05) | 347 | 15.24 (0.09) | 324 | 15.26 (0.10) | 671 | 15.25 (0.07) |
| Nasal ala length (mm) ^a^ | 937 | 65.90 (0.31) | 1504 | 61.24 (0.16) | 2441 | 63.03 (0.16) | 347 | 49.48 (0.21) | 324 | 48.86 (0.25) | 671 | 49.18 (0.16) |
| Nasal height (mm) | 939 | 54.23 (0.20) | 1508 | 53.38 (0.13) | 2447 | 53.71 (0.11) | 347 | 41.23 (0.19) | 324 | 40.50 (0.22) | 671 | 40.88 (0.14) |
| Nasal bridge length (mm) | 939 | 47.01 (0.21) | 1508 | 45.95 (0.13) | 2447 | 46.36 (0.11) | 347 | 34.27 (0.17) | 324 | 33.74 (0.20) | 671 | 34.01 (0.13) |
| Labial fissure width (mm) | 938 | 47.91 (0.20) | 1507 | 46.74 (0.12) | 2445 | 47.19 (0.11) | 347 | 41.20 (0.15) | 324 | 39.98 (0.16) | 671 | 40.61 (0.11) |
| Philtrum length (mm) | 939 | 15.61 (0.09) | 1507 | 13.97 (0.06) | 2446 | 14.60 (0.06) | 347 | 14.15 (0.07) | 324 | 13.49 (0.07) | 671 | 13.84 (0.05) |
| Upper lip height (mm) | 938 | 21.68 (0.10) | 1506 | 20.09 (0.07) | 2444 | 20.70 (0.06) | 347 | 19.74 (0.09) | 324 | 19.00 (0.10) | 671 | 19.39 (0.07) |
| Lower lip height (mm) | 925 | 18.37 (0.10) | 1503 | 17.27 (0.06) | 2428 | 17.69 (0.06) | 347 | 15.37 (0.08) | 324 | 15.13 (0.09) | 671 | 15.25 (0.06) |

^a^ mean values for these variables represent left and right measurements summed.


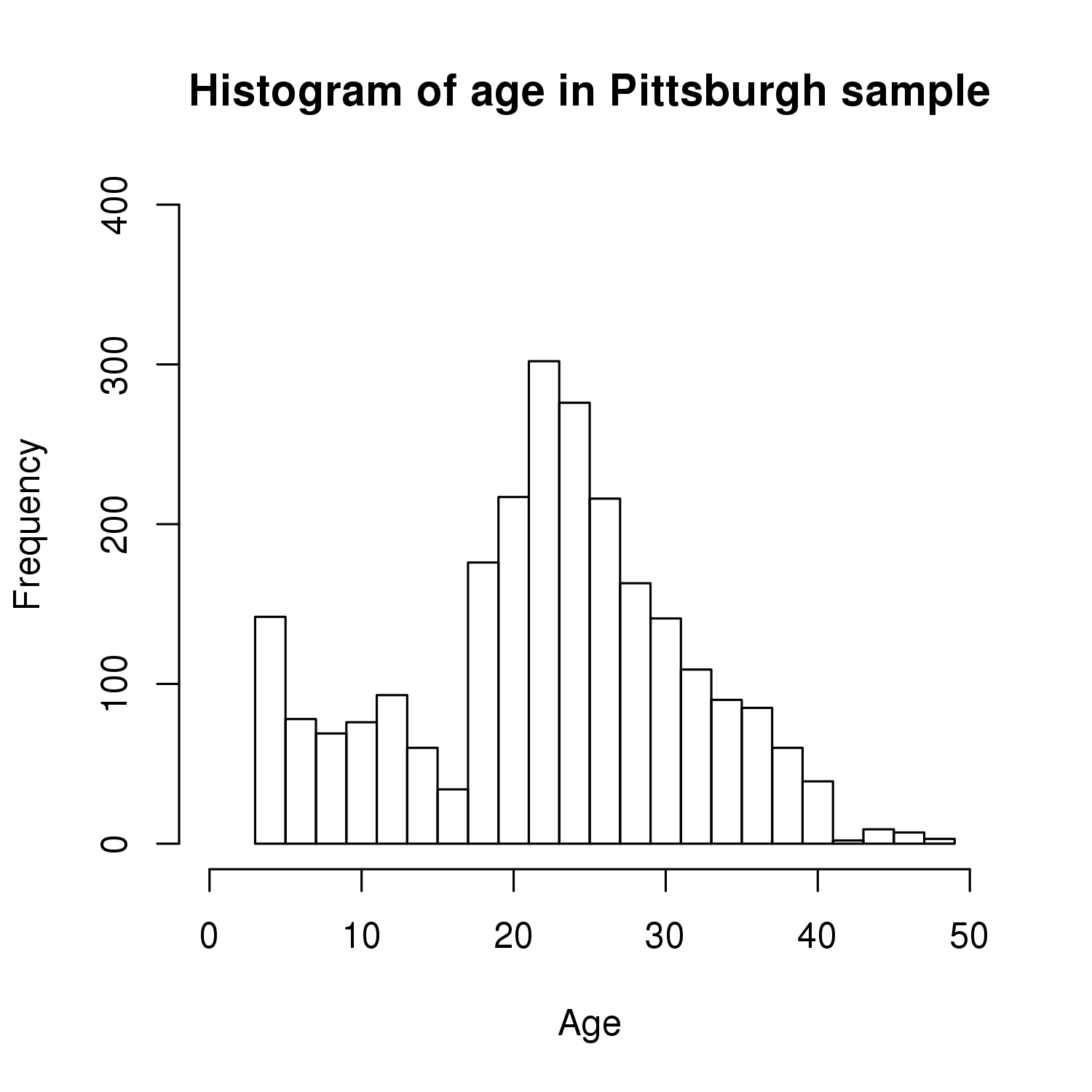

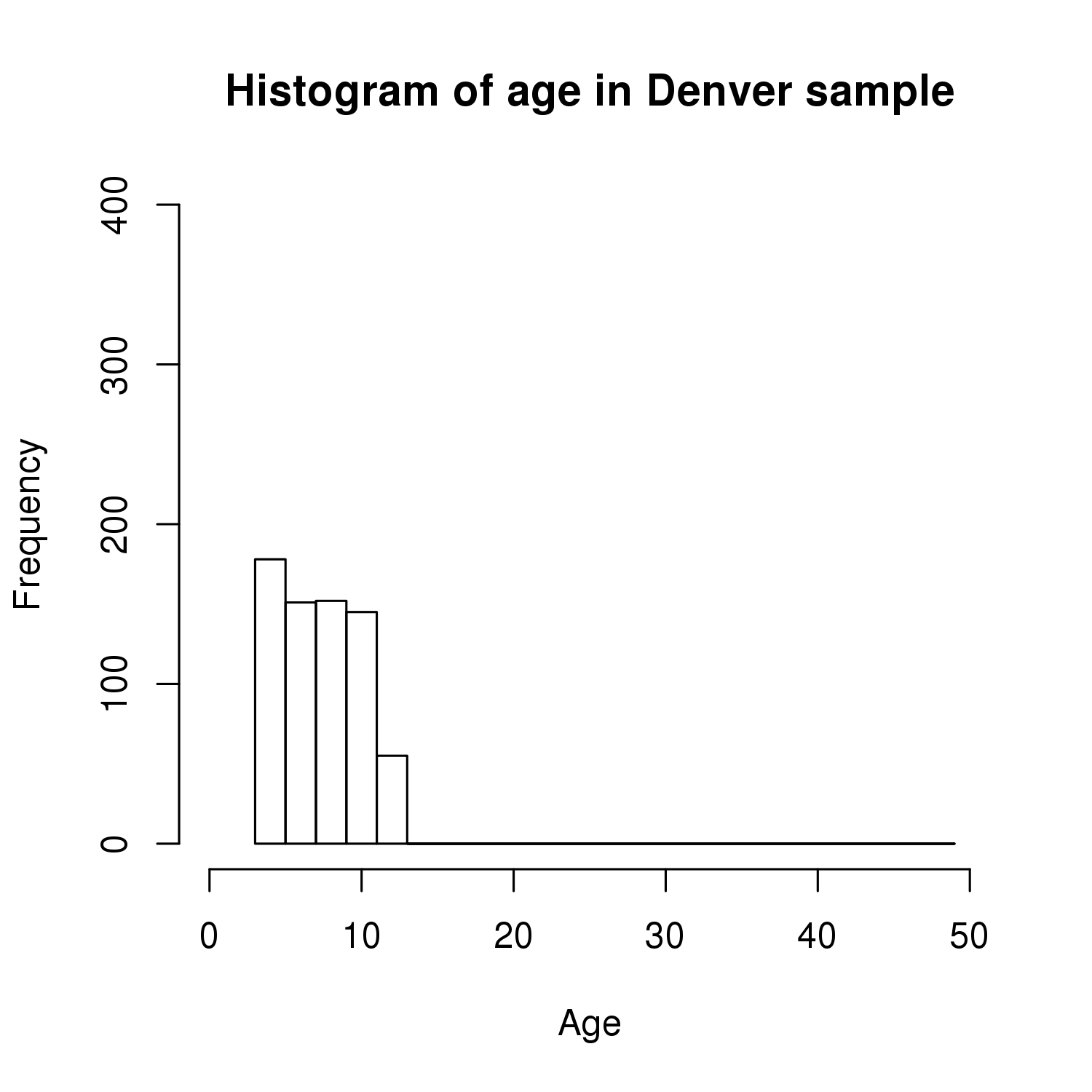

Supplement: S4 Table — (DOCX) [file pgen.1006149.s004.docx]
