# Supplementary material for: Genome-Wide Association Study Reveals Multiple Loci Influencing Normal Human Facial Morphology
Source: PLoS Genet. 2016 Aug 25;12(8):e1006149. doi: 10.1371/journal.pgen.1006149 (PMC4999139; doi:10.1371/journal.pgen.1006149)
Supplement: S5 Table — (DOCX) [file pgen.1006149.s005.docx]

S5 Table. List of linear distance measurements.

| **Measurement** | **Figure ^a^** | **Region** | **Landmarks involved** |
| --- | --- | --- | --- |
| Cranial base width | Fig 1A | Head | Right Tragion (t_r) - Left Tragion (t_l) |
| Upper facial depth ^b^ | Fig 1B | Face | Nasion (n) - Left Tragion (t_l) |
| Middle facial depth ^b^ | Fig 1C | Face | Subnasale (sn) - Left Tragion (t_l) |
| Lower facial depth ^b^ | Fig 1D | Face | Gnathion (gn) - Left Tragion (t_l) |
| Morphological facial height | Fig 1E | Face | Nasion (n) - Gnathion (gn) |
| Upper facial height | Fig 1F | Face | Nasion (n) - Stomion (sto) |
| Lower facial height | Fig 1G | Face | Subnasale (sn) - Gnathion (gn) |
| Intercanthal width | Fig 1H | Eye | Right Endocanthion (en_r) - Left Endocanthion (en_l) |
| Outercanthal width | Fig 1I | Eye | Right Exocanthion (ex_r) - Left Exocanthion (ex_l) |
| Palpebral fissure length ^b^ | Fig 1J | Eye | Left Endocanthion (en_l) - Left Exocanthion (ex_l) |
| Nasal width | Fig 1K | Nose | Right Alare (al_r) - Left Alare (al_l): |
| Subnasal width | Fig 1L | Nose | Right Subalare (sbal_r) - Left Subalare (sbal_l) |
| Nasal protrusion | Fig 1M | Nose | Subnasale (sn) - Pronasale (prn) |
| Nasal ala length ^b^ | Fig 1N | Nose | Left Alar Curvature Point (ac_l) - Pronasale (prn) |
| Nasal height | Fig 1O | Nose | Nasion (n) - Subnasale (sn) |
| Nasal bridge length | Fig 1P | Nose | Nasion (n) - Pronasale (prn) |
| Labial fissure width | Fig 1Q | Mouth | Right Chelion (ch_r) - Left Chelion (ch_l) |
| Philtrum length | Fig 1R | Mouth | Subnasale (sn) - Labiale Superius (ls) |
| Upper lip height | Fig 1S | Mouth | Subnasale (sn) - Stomion (sto) |
| Lower lip height | Fig 1T | Mouth | Stomion (sto) - Sublabiale (sl) |

^a^ Measurements shown in Figure 1, parts A-T

^b^ indicates bilateral measurements. Further definitions can be found here: <https://www.facebase.org/facial_norms/notes/>
