# Supplementary material for: Genome-Wide Association Study Reveals Multiple Loci Influencing Normal Human Facial Morphology
Source: PLoS Genet. 2016 Aug 25;12(8):e1006149. doi: 10.1371/journal.pgen.1006149 (PMC4999139; doi:10.1371/journal.pgen.1006149)
Supplement: S6 Table — (DOCX) [file pgen.1006149.s006.docx]

S6 Table. Number of SNPs omitted and retained for each quality filter

| **Step** | **Filter** | **SNPs omitted** | **SNPs cumulatively retained** |
| --- | --- | --- | --- |
| 1 | None (all SNPs) |  | 968,515 |
| 2 | CIDR technical filters | 8,470 | 960,045 |
| 3 | Missing call rate ≥ 2% | 9,675 | 950,370 |
| 4 | >1 discordant calls in 69 duplicates ^a^ | 26 | 950,344 |
| 5 | >1 Mendelian error across 8 HapMap trios | 122 | 950,222 |
| 6 | HWE p-value < 10^-4^ | 2,038 | 948,184 |
| 7 | Allele frequency difference ≥ 0.2 between sexes ^b^ | 274 | 947,910 |
| 8 | Heterozygosity difference ≥ 0.3 between sexes ^b^ | 41 | 947,869 |
| 9 | Positional duplicates | 19,597 | 928,272 |
| 10 | Monomorphic (MAF = 0) | 108,485 | 819,787 |
| 11 | MAF < 0.00621(Pittsburgh) ^c^  MAF < 0.02288 (Denver) ^c^ | 159,832  181,015 | 659,955  638,772 |

^a^ one duplicate was removed from QC filters due to a chromosomal anomaly

^b^ filter applied to SNPs on autosomes and XY pseudo-autosomal region

^c^ 2 x MAF x (1-MAF) x N > 30 (Pittsburgh N=2447, Denver N=671)
