# Supplementary material for: Genome-Wide Association Study Reveals Multiple Loci Influencing Normal Human Facial Morphology
Source: PLoS Genet. 2016 Aug 25;12(8):e1006149. doi: 10.1371/journal.pgen.1006149 (PMC4999139; doi:10.1371/journal.pgen.1006149)
Supplement: S7 Table — (DOCX) [file pgen.1006149.s007.docx]

S7 Table. Final number of SNPs used in our analysis

|  | **Genotyped** | **Imputed** | **Total** |
| --- | --- | --- | --- |
| Pittsburgh | 659,955 | 10,031,491 | 10,691,446 |
| Denver | 638,772 | 7,992,779 | 8,631,551 |
| Meta-Analysis | 637,391 | 7,922,324 | 8,559,716 |

Filters: Missing call rate ≥ 2%, HWE p-value<10^-4^, MAF<0.00621(Pitt), MAF<0.02288(Denver)

2 x MAF x (1-MAF) x N > 30 (Pittsburgh N=2447, Denver N=671)
