# Supplementary material for: Genome-Wide Association Study Reveals Multiple Loci Influencing Normal Human Facial Morphology
Source: PLoS Genet. 2016 Aug 25;12(8):e1006149. doi: 10.1371/journal.pgen.1006149 (PMC4999139; doi:10.1371/journal.pgen.1006149)
Supplement: S16 Fig — (A) meta-analysis results, (B) Pittsburgh sample results, and (C) Denver sample results. Lines for p-value thresholds set at 5 x 10^−8^ for genome-wide significance and 5 x 10^−7^ for suggestive significance. (PDF) [file pgen.1006149.s023.pdf]

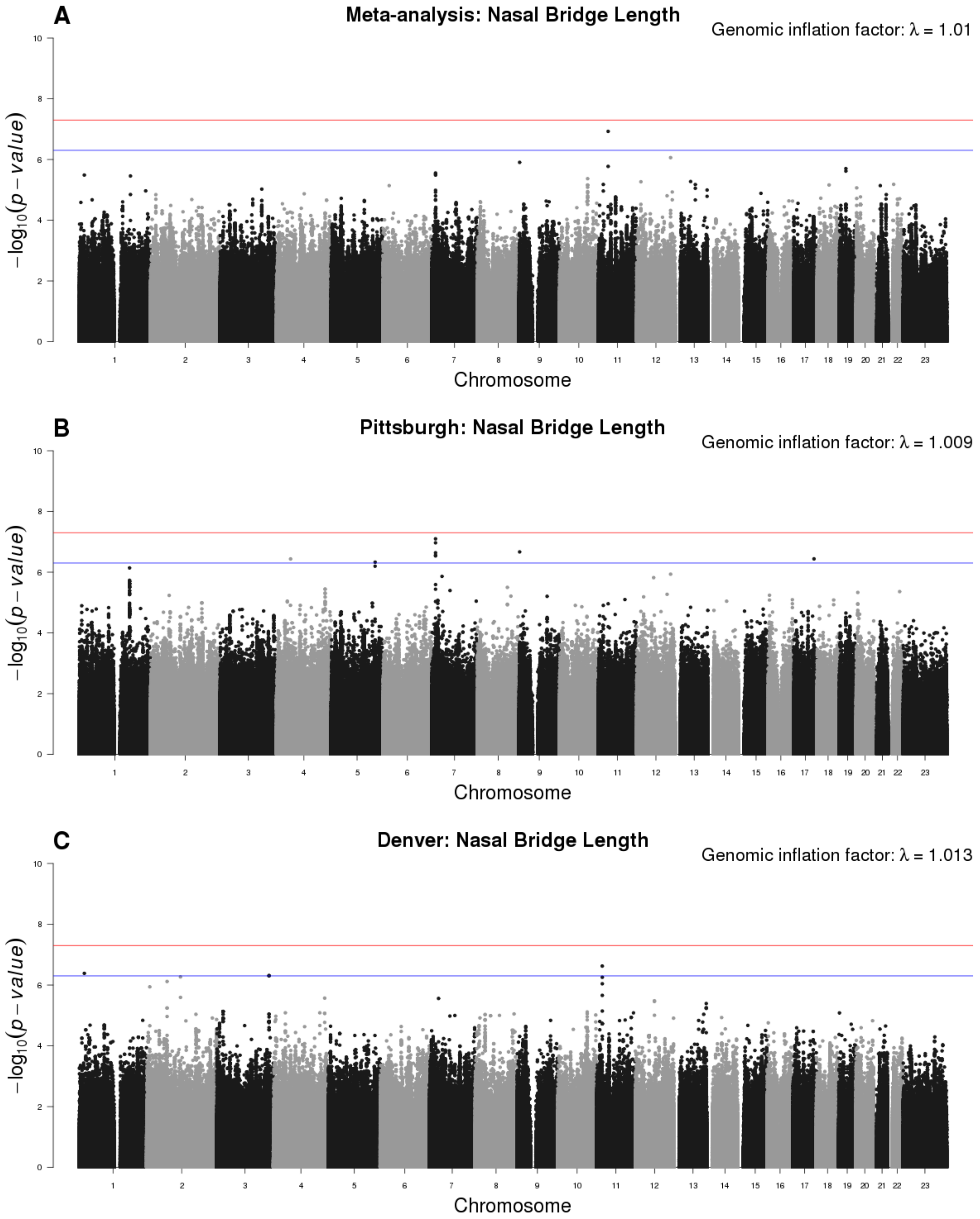

S16 Fig. Manhattan plots for nasal bridge length. (A) meta-analysis results, (B) Pittsburgh sample results, and (C) Denver sample results. Lines for p-value thresholds set at 5 x 10^{-8} for genome-wide significance and 5 x 10^{-7} for suggestive significance.
