# Supplementary material for: Genome-Wide Association Study Reveals Multiple Loci Influencing Normal Human Facial Morphology
Source: PLoS Genet. 2016 Aug 25;12(8):e1006149. doi: 10.1371/journal.pgen.1006149 (PMC4999139; doi:10.1371/journal.pgen.1006149)
Supplement: S21 Fig — LocusZoom plots comparing the (A) discovery analysis, and (B) conditional analysis for the observed genetic association of nasal width near PAX1 at 20p11.22. Genetic association (left y-axis; log10-transformed p-values) is shown for genotyped SNPs depicted as stars and imputed SNPs depicted as circles. Shading of the points represent the linkage disequilibrium (r2) between each SNP and the rs2424399 (the top SNP from the discovery analysis), indicated by purple shading. The blue overlay shows the recombination rate (right y-axis). Positions of genes are shown below the plot. In the discovery analysis, a possible second peak in low-LD with the rs2424399 was observed around chromosomal position 22.0 Mb. After conditioning on rs2424399, variants at position 22.0 Mb showed some independent evidence of association, although not meeting genome-wide or suggestive thresholds for significance. (PDF) [file pgen.1006149.s028.pdf]

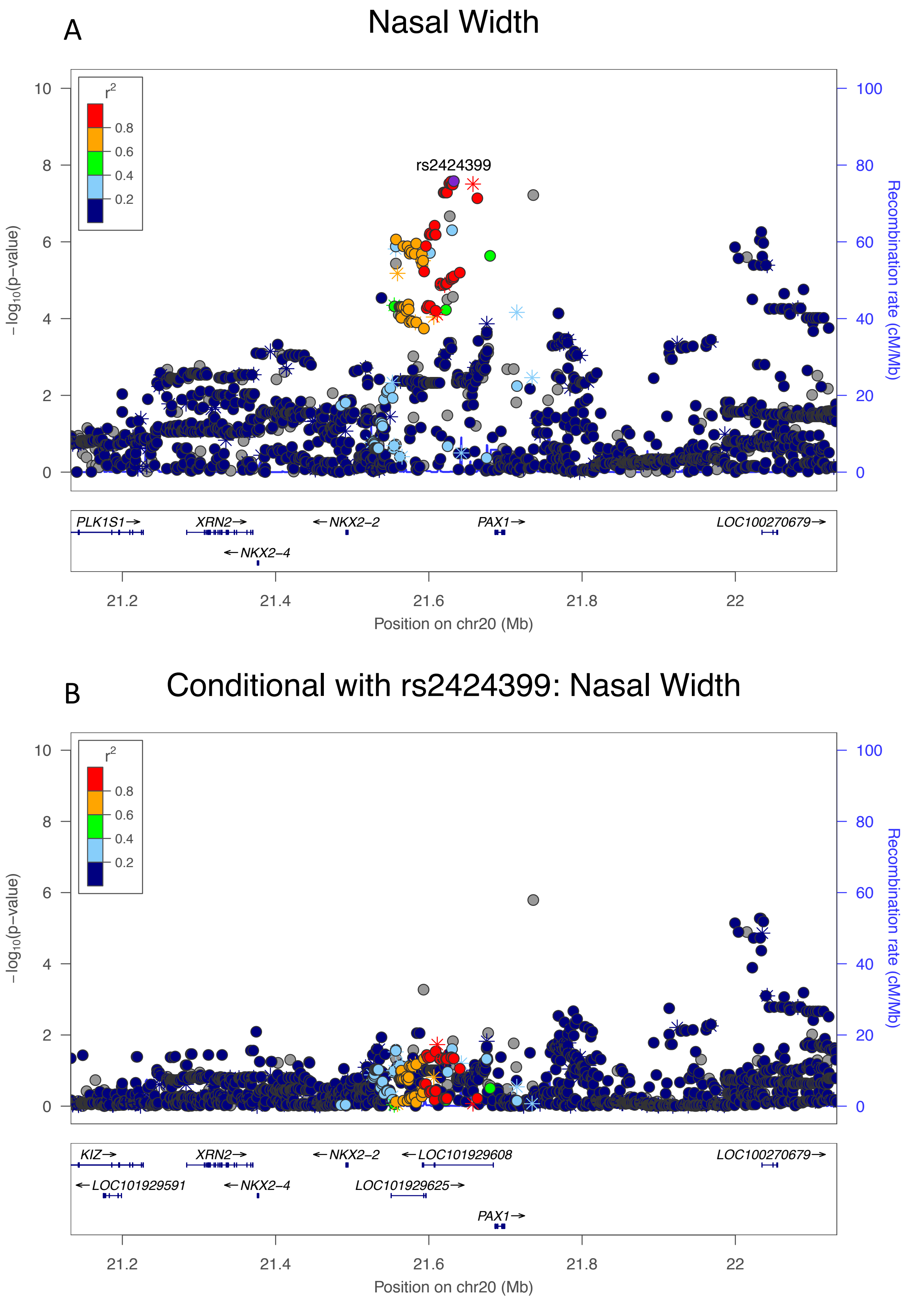

S21 Fig. LocusZoom plots comparing the (A) discovery analysis, and (B) conditional analysis for the observed genetic association of nasal width near PAX1 at 20p11.22. Genetic association (left y-axis; log10-transformed p-values) is shown for genotyped SNPs

depicted as stars and imputed SNPs depicted as circles. Shading of the points represent the linkage disequilibrium (r2) between each SNP and the rs2424399 (the top SNP from the discovery analysis), indicated by purple shading. The blue overlay shows the recombination rate (right y-axis). Positions of genes are shown below the plot. In the discovery analysis, a possible second peak in low-LD with the rs2424399 was observed around chromosomal position 22.0 Mb. After conditioning on rs2424399, variants at position 22.0 Mb showed some independent evidence of association, although not meeting genome-wide or suggestive thresholds for significance
