# Supplementary material for: Genome-Wide Association Study Reveals Multiple Loci Influencing Normal Human Facial Morphology
Source: PLoS Genet. 2016 Aug 25;12(8):e1006149. doi: 10.1371/journal.pgen.1006149 (PMC4999139; doi:10.1371/journal.pgen.1006149)
Supplement: S22 Fig — Landmarks shown in frontal view (A) are n = nasion; prn = pronasale; sn = subnasale; ls = labiale superius; sto = stomion; li = labiale inferius; sl = sublabiale; gn = gnathion; en = endocanthion; ex = exocanthion; al = alare; sbal = subalare; cph = crista philtra; ch = chelion (for bilateral points only right side labeled). Landmarks shown in the lateral view (B) are ac = alar curvature point and t = tragion (only left landmark shown for these two bilateral points). (PDF) [file pgen.1006149.s029.pdf]

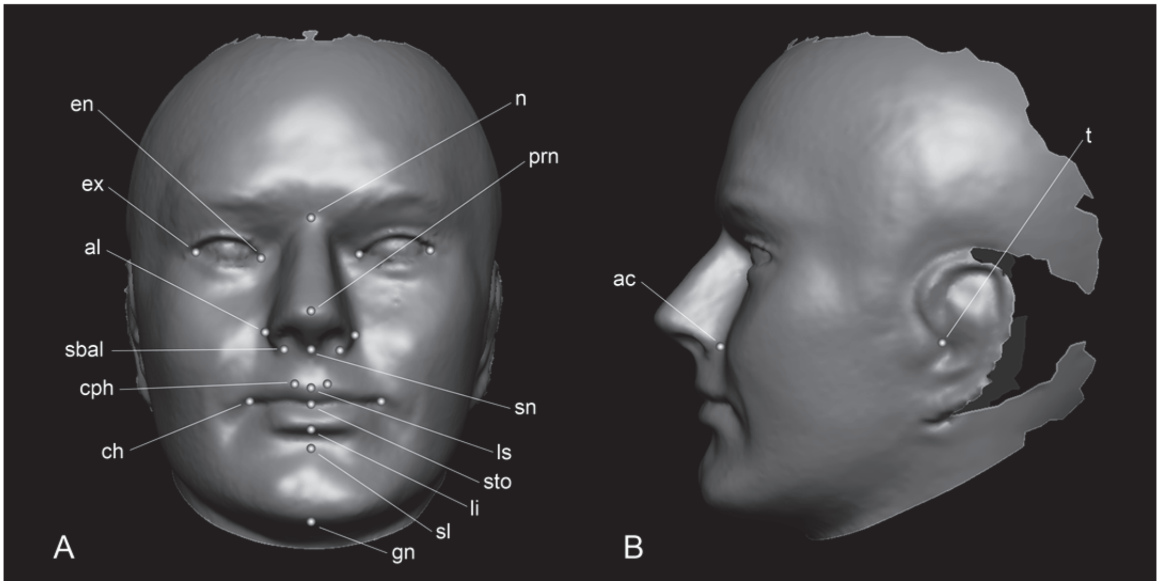

S22 Fig. 3D facial surface model showing the location of the 24 standard landmarks used to generate the linear distances. Landmarks shown in frontal view (A) are n = nasion; prn = pronasale; sn = subnasale; ls = labiale superius; sto = stomion; li = labiale inferius; sl = sublabiale; gn = gnathion; en = endocanthion; ex = exocanthion; al = alare; sbal = subalare; cph = crista philtra; ch = chelion (for bilateral points only right side labeled). Landmarks shown in the lateral view (B) are ac = alar curvature point and t = tragion (only left landmark shown for these two bilateral points).
