# Supplementary material for: Genome-Wide Association Study Reveals Multiple Loci Influencing Normal Human Facial Morphology
Source: PLoS Genet. 2016 Aug 25;12(8):e1006149. doi: 10.1371/journal.pgen.1006149 (PMC4999139; doi:10.1371/journal.pgen.1006149)
Supplement: S23 Fig — The proportion of total genetic variation explained by each principal component of ancestry is indicated on the axis. (PDF) [file pgen.1006149.s030.pdf]

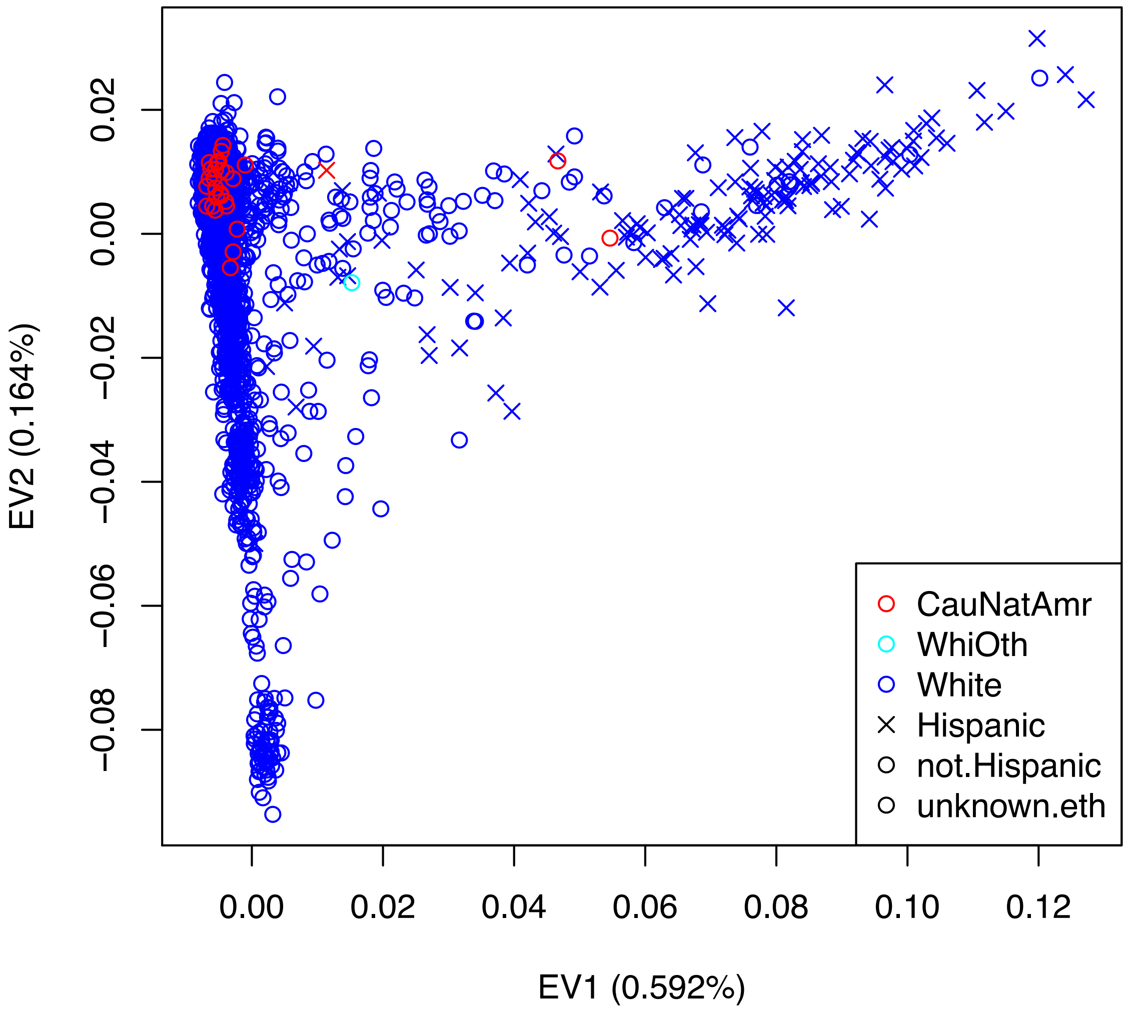

S23 Fig. Plot showing population stratification across the first two principal components of ancestry (EV1 and EV2). The proportion of total genetic variation explained by each principal component of ancestry is indicated on the axis.
